# Unveiling scientific articles from paper mills with provenance analysis

**DOI:** 10.1371/journal.pone.0312666

**Published:** 2024-10-30

**Authors:** João Phillipe Cardenuto, Daniel Moreira, Anderson Rocha

**Affiliations:** 1 Artificial Intelligence Lab. Recod.ai, Institute of Computing, Universidade Estadual de Campinas, Campinas, São Paulo, Brazil; 2 Department of Computer Science, Loyola University Chicago, Chicago, Illinois, United States of America; Universidade de São Paulo, BRAZIL

## Abstract

The increasing prevalence of fake publications created by paper mills poses a significant challenge to maintaining scientific integrity. While integrity analysts typically rely on textual and visual clues to identify fake articles, determining which papers merit further investigation can be akin to searching for a needle in a haystack, as these fake publications have non-related authors and are published on non-related venues. To address this challenge, we developed a new methodology for provenance analysis, which automatically tracks and groups suspicious figures and documents. Our approach groups manuscripts from the same paper mill by analyzing their figures and identifying duplicated and manipulated regions. These regions are linked and organized in a provenance graph, providing evidence of systematic production. We tested our solution on a paper mill dataset of hundreds of documents and also on a larger version of the dataset that deliberately included thousands of documents intentionally selected to distract our method. Our approach successfully identified and linked systematically produced articles on both datasets by pinpointing the figures they reused and manipulated from one another. The technique herein proposed offers a promising solution to identify fraudulent manuscripts, and it could be a valuable tool for supporting scientific integrity.

## 1 Introduction

In 2018, Jana Christopher raised concerns about the systematic and large-scale fabrication of image results in biomedical manuscripts [1]. While working for the Federation of European Biochemical Societies’ Press (FEBS Press), she reported sets of papers formally submitted to peer review containing suspicious Western blots. The unveiled problem resided mostly on the recurrence of unrelated experimental outcomes presenting identical substrate backgrounds and too similar individual bands. This suggested the probable composition of images by splicing together the same set of Western blots onto the same empty background to support ungrounded results. The involved publications —all rejected by FEBS Press— did not present an obvious relation regarding authorship or authors’ affiliation.

Elisabeth Bik and other investigators later confirmed Christopher’s concerns by reporting an extensive collection of manuscripts allegedly belonging to *paper mills* [2] (*i.e.*, undisclosed actors who seem to systematically fabricate scientific articles and forge images to support the articles’ claims, regardless of the absence of scientific ground). As of March 2021, Bik *et al.* have listed more than 1,300 documents suspected of coming from paper mills, of which 370 were retracted to date [3].

Paper mills pose a new challenge to the community of scientific integrity verification. Typical cases of scientific misconduct comprise the inappropriate reuse, duplication, or manipulation of images executed by the same group of researchers, who usually split paper authorship and belong to the same laboratory [4]. On the contrary, paper mills are the source of several suspicious manuscripts that, despite sharing fabricated content, do not present a relation in authorship or authors’ affiliation [1]. Consequently, fake articles from paper mills may appear in diverse venues with non-related authors and topics, thus generating a needle-in-the-haystack problem. This new challenge is changing the scientific integrity landscape, forcing the community to rethink its guidelines and detection tools [5].

The dangers posed by paper mills are not yet fully comprehended, but their immediate effects can potentially contaminate scientific literature with unreliable information. This information, if cited as scientific evidence by other honest studies, could erode trust in science [6]. An even more alarming concern is the false connections between human genes and specific cancer types suggested by these fake articles. This could harm the field of human genes and potentially provide misleading scientific support for cancer research, wasting crucial time and grant funds on irreproducible experiments performed by careful scientists [7].

**Fig 1:**
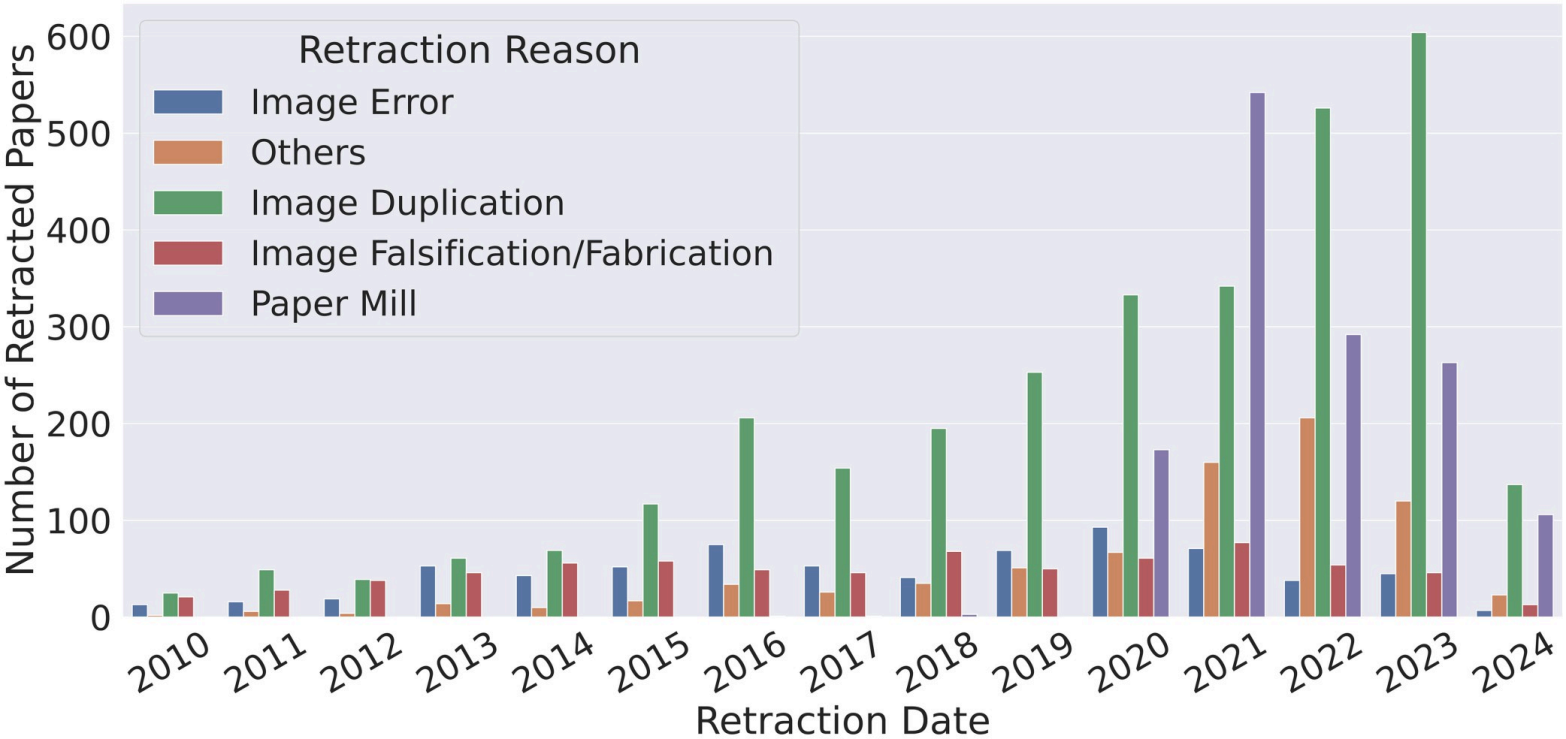
Retracted papers due to image problems from 2010 to 2024. Paper mill production has drastically increased from 2020 to 2024. The category “Others” regards ambiguous retraction reasons related to images that we could not fit into any other category.

To understand the relevance of paper mills in the context of scientific integrity and problems related to images, we inspected the reasons for article retraction involving controversial images from 2010 to 2024 according to the Retraction Watch database [8]. As one might observe in Fig 1, the systematic fabrication of suspect papers has been a prevalent issue since 2020 and comprises 20% of the retraction due to image concerns. We group this same data by scientific area in Table 1. Biomedical articles concentrate most of the retractions (90%), encompassing almost all the cases of paper mills. Based on this scenario, we decided to focus on biomedical manuscripts, planning to expand to other areas in the future.

**Table 1:** Retractions due to problematic images by scientific area from 2010 to 2024.

Area	#Retracted Papers	#Paper Mill Retractions
**Biomedical**	6046	1376
**Physics**	524	1
**Business**	38	0
**Social Sciences**	32	4
**Environ. Sciences**	25	0

### 1.1 Data source

Retraction Watch database as of May 2024.

Our main research endeavor is pursuing an understanding of paper mills’ operations, seeking an answer to “How to identify paper mill productions within a large collection of suspect articles?” As a research outcome, we propose a new forensic method extending provenance analysis [9] to the case of biomedical scientific articles.

Provenance analysis, in essence, is a technique employed to track the different versions of a media asset within a database [9]. In our specific case, we employ provenance analysis to trace the reuse of different versions of a scientific image across articles produced by paper mills. Originally, image provenance analysis was designed for natural photographs [9], such as those found in social media, but it does not conform to the unique characteristics of scientific imagery (*e.g.*, microscopic material, and Western blots, instead of natural scenes and everyday objects). Because of this disparity, we extend and adapt provenance analysis specifically for the scientific domain.

The proposed method starts with a collection of thousands of articles (*i.e.,* the haystack) in Portable Document Format (PDF) and ends with pinpointing the most problematic figures within their suspicious documents (*i.e.,* the needles). We rely on forensic image artifacts to link the figures—and ultimately the documents—that share content. Forensic image artifacts are distinct and identifiable traces within images that provide clues for their source content. These artifacts can be identified by sensor noise analysis (*e.g.,* photo-response non-uniformity noise), object descriptors, and deep neural network-derived features [10].

We test our technique on a set of publications suspected of coming from paper mills, reported by Bik [11]. Dr. Bik named this dataset the Stock Photos Paper Mill (SPP). In addition, we also test the solution in an extended version of SPP, to which we have added thousands of *distractor* documents (*i.e.*, papers without known issues). The extended SPP helps us understand the solution’s performance in a more realistic needle-in-the-haystack scenario, where there is a predominance of authentic articles and a smaller group of problematic ones. Our experiments demonstrate that the proposed method can efficiently and effectively unveil suspicious relationships among documents in large-scale scenarios of thousands of articles, grouping them according to the category of the reused images (*e.g.*, microscopy imaging).

### 1.2 Disclaimer

Although we rely on the impressive work of Bik and collaborators, who disclosed and collected the potential problems of the papers belonging to the SPP dataset, we must highlight that the proposed solution can neither establish the intentions of the authors of these papers nor it is our purpose to judge or denounce their actions. From our standpoint, the mere presence of an article within SPP (and its extended version) does not mean the presence or absence of misconduct. We are simply finding potential shared content between articles. Our ultimate goal is to make this tool available to the community and officials from institutions and integrity offices, who will have the final word about the cases.

In summary, the contributions of this work are:

- A novel automated solution to the problem of unveiling scientific articles suspected of coming from paper mills by relying on the articles’ figures. The proposed method starts by automatically extracting figures from thousands of PDF articles and ends with a rich visualization of the shared content at both the figure and document levels.
- A new provenance analysis method tailored for biomedical images that can track reused images throughout multiple publications.
- An annotated dataset composed of scientific articles suspected of coming from paper mills added to distractor publications (*i.e.*, regular papers without known problems). This dataset is an extension of Bik’s work [11], containing 4,869 scientific documents, of which 121 (∼2.5%) were documented as suspects of belonging to mills. We report quantitative and qualitative results of the proposed technique and two other baselines from the literature over this dataset.
- A machine learning-based content extractor of biomedical scientific figures, which segments the compound figures into multiple independent panels for further analyses. The extractor also filters the panels according to their type (*e.g.,* microscopy imaging, blots), prioritizing the types with a high prevalence of misuse, according to scientific integrity analysts [4].

The remainder of the article is structured into the following sections: Sec. 2 presents the related works, followed by Sec. 3, which details the proposed method. Sec. 4 presents the experimental settings, and Sec. 5 presents the results. Finally, Sec. 6 encompasses the conclusions and outlines future work directions.

## 2 Related works

Digital forensics is crucial for maintaining scientific image integrity in the digital age. Early forensic tools have focused on detecting scientific image tampering, using image segmentation techniques as proposed by Farid [12] and pixel-wise comparison as proposed by Koppers *et al.* [13]. However, with the increase in image reuse and manipulation across different scientific articles, new automated methods have emerged to address these issues, *e.g.,* Bucci [14]. Furthermore, sophisticated forensic techniques that leverage artificial intelligence have been proposed to detect scientific image tampering, such as MONet [15]. While these methods have shown some effectiveness, further research and development are still needed to improve their performance and make them more robust for scientific integrity assessment [16].

To our knowledge, no digital forensic technique has been proposed using provenance analysis to detect paper mills. Additionally, research is lacking in analyzing large datasets for scientific image integrity. One of the most well-known works that studies large-scale scientific image integrity is Acuna *et al.* [17], which uses a human-assisted methodology to identify image reuse in the bioscience scale. Acuna *et al.*’s solution starts with a copy-move detector across figures, followed by an automated model that classifies the matched image pair as biological or not. Finally, a human revises the biological matched pairs, looking for problematic reuse. Although they tested their method on 2.7 million scientific figures, their analysis was limited to figures from the same first and last authors in the collection. Consequently, an image would only be compared to a few hundred others, *i.e.*, the other figures from the same authors. While their method represents an advance for scientific image integrity, the authors acknowledge that their approach is computationally complex and not scalable for comparing thousands of images.

A more complex tool for scientific image analysis was proposed by Moreira *et al.* [18] using digital forensic-based state-of-the-art techniques. Their system can deal with real-world cases, starting from PDF documents and ending with copy-move detection and provenance analysis of the figures. Their provenance solution, which is a combination of Moreira *et al.* [19] and Bharati *et al.* [20], presents the first solution that leverages the provenance analysis pipeline for scientific image integrity to the best of our knowledge.

A similar task employed in digital forensics for finding reused and manipulated images is copy-editing detection. Similarly to the provenance task, copy-editing detection relies on two stages: retrieval and verification. In the retrieval stage, a method generates a list of candidate images that may have reused the content of a query. During the verification stage, the method assesses whether the content of each candidate image matches the query. However, different from provenance analysis, the purpose of copy-editing detection is not to trace the origin of an image but solely to identify potential instances of image reuse.

The state-of-the-art copy-editing retrieval task is Self-Supervised Descriptor for Image Copy Detection (SSCD) [21], in which a self-supervised machine learning technique is employed to acquire robust features geared towards enhancing the retrieval stage. For training their solution, the authors leveraged DISC [22], a dataset with more than a million images, including 50,000 query images, of which 10,000 are true copies. It is worth noting that while SSCD has demonstrated remarkable results, it comes with a considerable computational cost and may not be suitable for deployment on conventional computers as the ones found on research integrity or publishers’ offices. Additionally, SSCD’s training dataset consists of natural images (commonly found on social media), and as such, it may need further refinement to align effectively with the scientific domain.

## 3 Method

The proposed method is structured into main stages organized in two sections: Sec. 3.1 *Filtering & Evidence Collection* and Sec. 3.2 *Scientific Content Provenance Analysis*. The first describes the proposed filtering process that allows our method to be applied to large datasets. During this stage, we extract the figures from PDF documents, identify and isolate their panels, and create deep-learning descriptions of each panel. The second stage performs a provenance analysis method tailored for scientific images on top of the extracted panels. Each stage is detailed in the following.

### 3.1 Filtering & evidence collection

**Fig 2:**
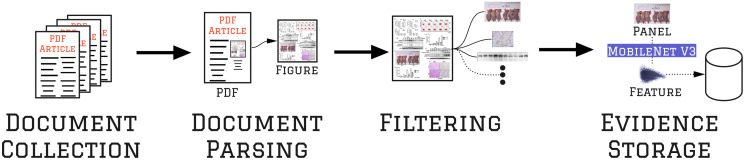
Filtering & evidence collection workflow. A suspect collection of documents undergoes parsing and figure extraction, resulting in a set of figures. These figures are then processed by a filtering stage that identifies and extracts the panels of interest. Later, a machine learning model [23] creates a robust evidence representation that can withstand transformations commonly applied to panels, such as resizing, compression, and color changes. Finally, each panel representation is stored in a database for further analysis. The figure used to depict this workflow is available under the Creative Commons license at https://doi.org/10.1371/journal.pone.0152712.g002.

The Filtering & Evidence Collection stage aims to parse, organize, and gather evidence for further analysis, as Fig 2 shows. The process begins with collecting PDF documents, from which all figures are extracted using an automated method (*Document Parsing*). The solution identifies and extracts each figure’s panels, filtering them into the five types of interest (*Filtering*). Finally, an artificial intelligence model is used to identify each image’s unique features, and their descriptions are saved in a database (*Evidence Storage*), which will be used in the *Provenance Analysis* phase to identify similar panels.

#### 3.1.1 Document parsing

Given the *Portable Document Format* (PDF) as the standard for scientific documents, we included an automated PDF figure extraction in the pipeline. Of course, this step is not required if it is possible to collect the scientific figures directly from another source. However, many online publications do not release their figures separately from their PDF document, making the scientific image analysis dependent on a PDF parser. For example, we experience this behavior in most articles from the SPP dataset, whose images are not available for download, only the PDF. To parse a PDF document and extract its figures, we use Moreira *et al.*’s PDF parser [18].

#### 3.1.2 Filtering

**Fig 3:**
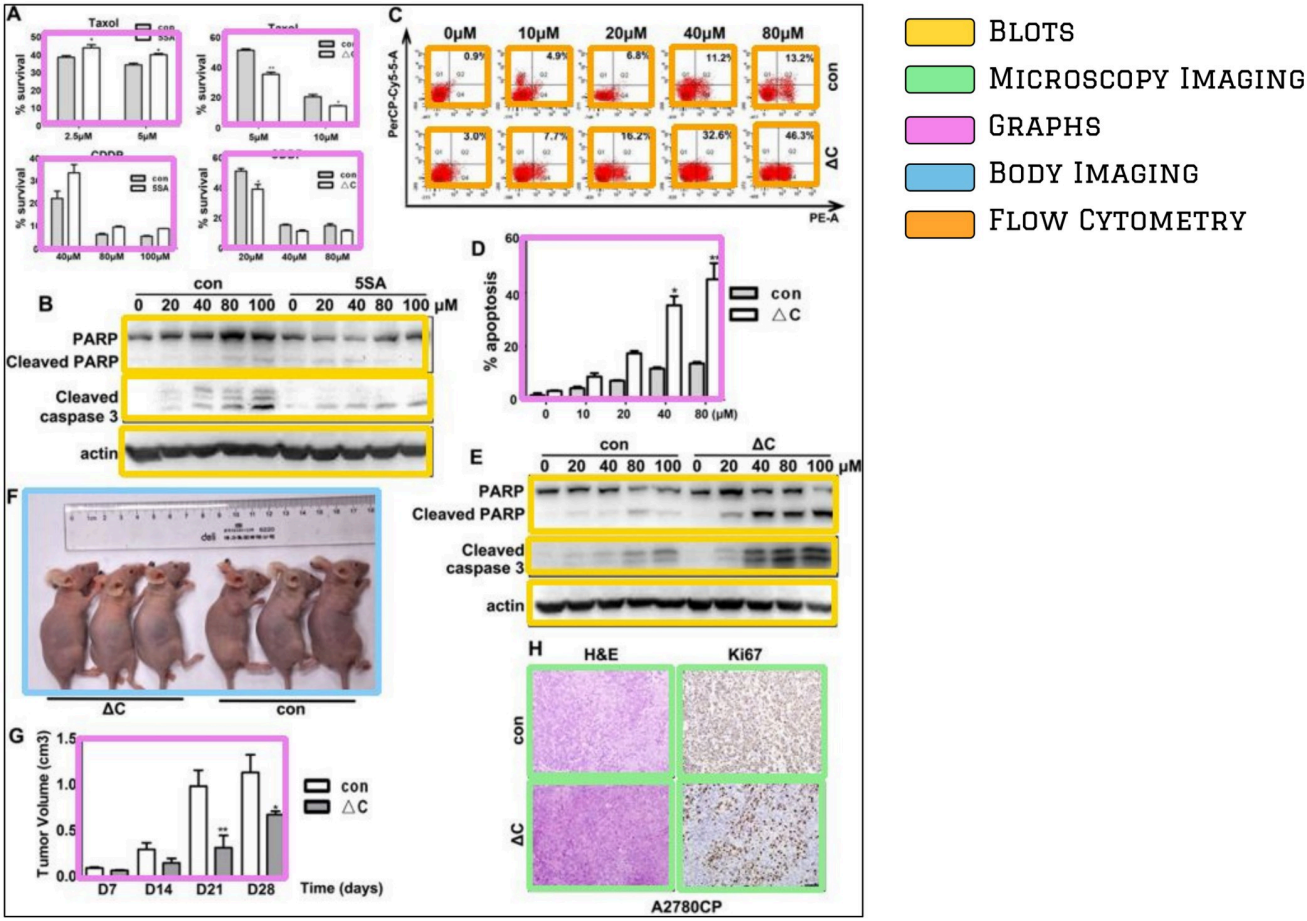
Compound figure annotation for panel extraction. Each colored rectangle corresponds to a panel that should be extracted as part of the panel extraction task. The categories of each rectangle are indicated by their color. For instance, the green panels are annotated as microscopy imaging. To generate this example, we used the figure distributed under a Creative Commons license found at https://doi.org/10.1371/journal.pone.0152712.g002.

Biomedical articles often contain complex figures with multiple panels that depict various analyses of an experiment (see Fig 3). However, certain regions within these figures may not provide informative content and could hinder image analysis due to their inherent similarities, such as letter-based labels or schematic diagrams. Filtering and retaining only the informative regions is crucial to reduce false-positive matches and ensure scientific image integrity. To identify these regions, we consulted recognized integrity guidelines and studies [1, 4, 24], which pointed to five commonly reported figure panel types often associated with image manipulation in Biomedical research. Therefore, the work herein focuses on the most frequently reported image panels despite the diversity in panel types and structures of scientific images. Fig 3 depicts a sample where each colored rectangle represents a panel type of interest. These panels are defined as:

- **Microscopy Imaging**: Photos taken by a microscope that can be fluorescent labeling, histology staining, or other types of tagging of cells or tissues, or components within, captured by light, electron, fluorescent, or other microscope types.
- **Blots**: The resulting image from techniques to detect specific proteins in a tissue sample or extract them using electrophoresis. This category includes Western, Northern, and Southern blots.
- **Body Imaging**: Image of the whole organ—from any living being—including images from Magnetic Resonance Imaging (MRI), Computerized Tomography/Computerized Axial Tomography (CT/CAT) scans, Positron Emission Tomography (PET), X-ray, and ultrasound.
- **Graphs and Plots**: Experimental data charts including bar, line, scatter, pie, area, and histogram plots.
- **Flow Cytometry**: The resulting plot from a technique used to detect and measure physical and chemical characteristics of a population of cells or particles.

The filtering stage employs an *Automated Panel Extraction* that uses a YoloV5 [25] deep learning model-based object detection method to locate and label the commonly reported panel types within an input figure. While other detection models could be used, we opted for a lightweight model like YoloV5 to ensure that our filtering stage remains computationally efficient and accessible to integrity analysts with limited computing resources.

The proposed filtering model was pre-trained on natural images—*e.g.,* social-media photos from the COCO [26] dataset—and later fine-tuned for scientific figures. For the pre-trained model, we used the weights from YOLOv5x6 provided by the YoloV5 authors [25]. To fine-tune this model on scientific data, we collected 3,836 biomedical article figures under a Creative Commons license, considering only those with at least one selected panel type (*e.g.,* Microscopy or the other four image types).

We annotated the articles’ figures using Label Studio [27] to locate the panels within them. Using such software, we can pinpoint the coordinates of the bounding boxes surrounding each image panel boundary and label each one for a different class, as presented in Fig 3. To obtain the figures, we used the Open Access Biomedical Image Search Engine’s API [28] and queried for the panel types of interest. We included additional information regarding the panel extraction solution in the S1 Appendix. The panel extraction implementation and model are freely available on the repository of this work.

#### 3.1.3 Evidence storage

While preparing figures for scientific articles, researchers often apply various post-processing operations, such as resizing, color adjustments, and compression, to enhance the visibility of their findings. However, these operations can inadvertently introduce artifacts or remove crucial information from the image. For example, even a simple photograph resizing to fit it into a panel placeholder can result in distortions or loss of important details, affecting the integrity analysis.

To avoid such artifacts distracting provenance analysis, we create evidence representations of each panel image content using a deep learning model robust to several types of image processing (*e.g.,* color-changing, noise, rescale, mirroring, crop, among others). This model’s robustness ensures that the original image and its processed versions (*e.g.,* a rescaled image) are similar. This allows the provenance analysis to retrieve the original image by giving a processed version and vice versa, all based on their visual similarity.

For creating these representations, we leverage the MobileNetV3 [23] model, which is pre-trained on ImageNet [29], a large dataset for image classification. MobileNetV3 is a lightweight and powerful model for image description that identifies robust features from an image using low computational power. To extract the evidence descriptions of each image, we get the feature vector from the last layer of MobileNetV3 after removing its classification portion. While MobileNetV3 has proven its effectiveness and efficiency for this purpose, it is crucial to acknowledge that the field of deep learning descriptors is rapidly evolving, and investigating more recent models tuned to the scientific domain might improve this stage.

### 3.2 Scientific content provenance analysis

Provenance analysis is a powerful technique for identifying and understanding object relationships within a collection, especially when suspicious links exist. One common application of provenance analysis is understanding the relationships between a single media item and a data collection [19]. In the herein work, we are interested in identifying potentially suspicious links between multiple scientific documents and their figures, which may indicate documents produced by a paper mill. To achieve this, we have divided provenance analysis into two levels: image and document. Both levels provide invaluable insights for analysts conducting investigations. The image level identifies the reuse and manipulation of panel images across a set of articles, while the document level visualizes the most suspicious systematically produced documents. While this study focuses on image content to reveal potential problematic connections between scientific papers, it is important to state that the articles’ texts, abstracts, titles, and other types of data (*e.g.,* gene sequences) could also be used to unveil paper mill traces [5, 11].

### 3.3 Provenance analysis at image level

Image provenance analysis tracks forensic clues that indicate image manipulation and duplication. Leveraging the collected figure panels extracted through the *Filtering & Evidence Collection* process, our method tracks reused panels and identifies possible manipulated versions.

**Fig 4:**
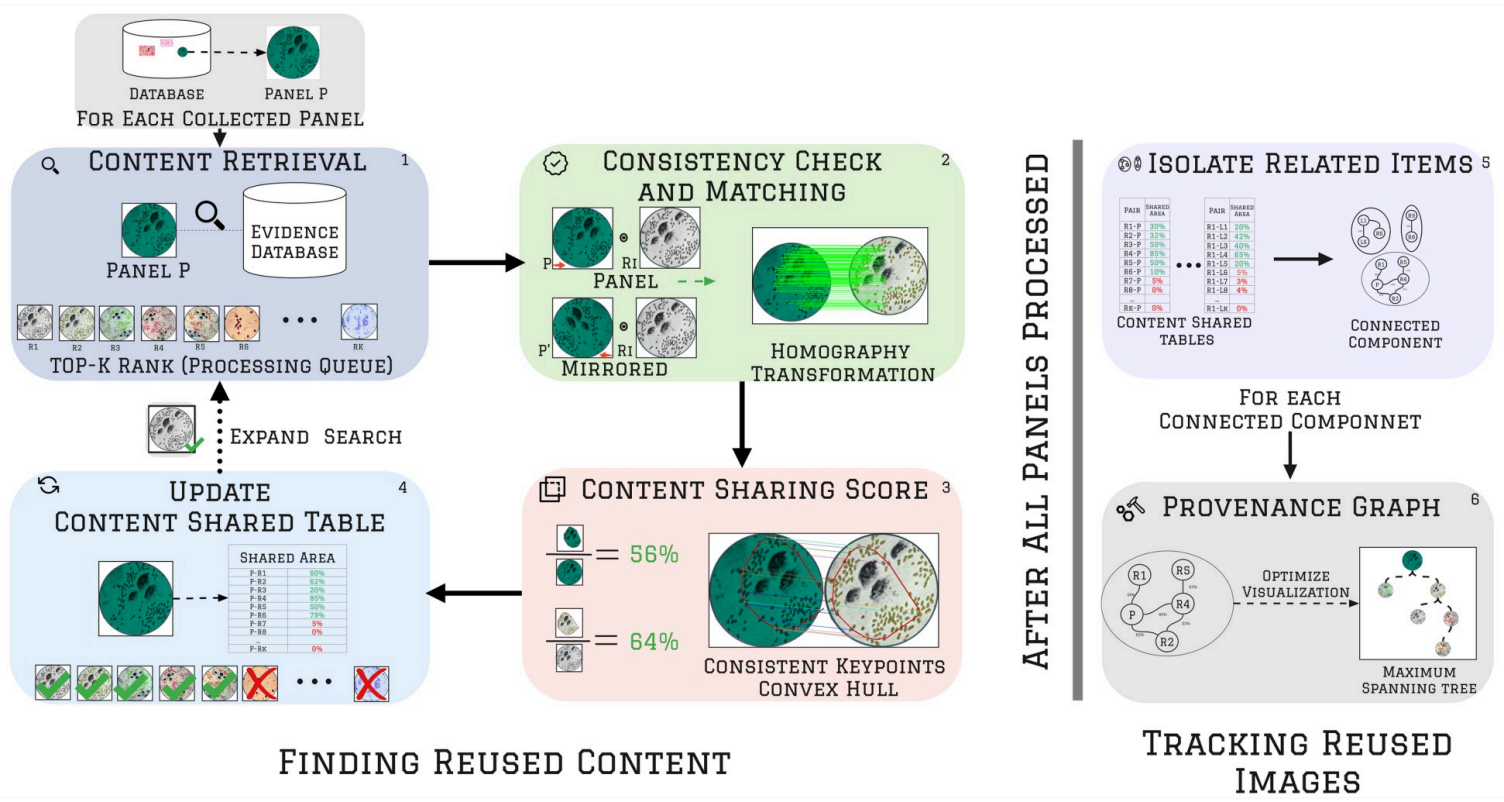
Provenance analysis at image level. Provenance analysis is performed for each panel collected during the *Filtering & Evidence Collection* step. Let *P* be one of these panels. First, the method performs a content retrieval by comparing the similarity of *P*’s description with the other panels in the database (step 1). The top-*K* similar panels to *P* are included in a processing queue. Then, the next panel *R*_*I*_ from the queue is compared to *P* to determine if they have consistent content (step 2). If *R*_*I*_ matches *P*, the method calculates the content-sharing score of *P* and *R*_*I*_ (step 3). This score informs the area shared between these panels. Using such a score, the method updates a content-shared table (step 4). If the content-sharing score is above a threshold (1%), the processing queue will expand with more *L* (*L* < = *K*) similar panels to *R*_*I*_. After processing all collected panels, the method starts constructing the provenance graph. It uses the scores located at the content-shared tables to identify the relationship of each pair of images within the collection. Then, it isolates the panels that relate to one another by finding their connected components (step 5). Finally, to visualize these components more clearly, the method generates provenance graphs by computing the maximum spanning tree of each connected component (step 6). This process results in a tree-like structure that shows the relationships between the panels within each connected component. The images from this figure were retrieved from a public domain source, in which we created multiple versions of the same image for illustration’s sake (https://pixnio.com/science/microscopy-images/tularemia-francisella-tularensis/photomicrograph-of-francisella-tularensis-bacteria-using-a-methylene-blue-stain).

The provenance workflow steps are divided into 1. *Content Retrieval*; 2. *Consistency Check and Matching*; 3. *Content Sharing Score Calculation*; 4. *Content Shared Table Building*; 5. *Identification of Suspicious Images*; and 6. *Provenance Graph Output*. Fig 4 depicts the complete analysis. For didactic sake, in the following, we describe the provenance workflow using a generic image *panel P* from the database returned by the *Filtering & Evidence Collection*. In practice, all other panels from the database will also be processed using the same workflow.

#### 3.3.1 Content retrieval

Provenance analysis starts by performing a content-based image retrieval using *P* as a query. This step is depicted in Fig 4 (step 1). To perform content retrieval, the method compares the features of *P* (extracted during the *Filtering & Evidence Collection* stage) with all panels of the same type of *P* (*e.g.,* all other Microscopy panels) within the database using cosine similarity. Then, the top-*K* similar panels to *P* (*K* = 400) are inserted into a processing queue. Panels extracted from the same source document as *P* are not included in the processing queue, as our goal is to identify reused and manipulated images across different documents.

During our experiments, we stored the similarity of each image’s feature on a matrix. However, for cases involving potentially millions of images, we recommend adopting more scalable solutions such as a vector database (*e.g.,* MilvusDB [30]) designed specifically for scalable similarity search.

#### 3.3.2 Consistency check and matching

This step compares each element from the queue with *P* using a local description strategy, which involves comparing the regions of *P* to the regions of the items from the queue at the pixel level. During this analysis, the method searches for reused regions and possible manipulation traces. Fig 4 (step 2) depicts this process. The local description analysis is computationally expensive, and comparing all elements in the database with *P* would take a significant amount of time (weeks or months for large databases). Content Retrieval plays a crucial role in the Provenance Analysis by reducing the local analysis of *P* to the top-*K* most similar panels in the database. In addition, this approach enables matching to be quickly accomplished (in minutes or seconds).

Let the next item from the processing queue be denoted as *R*_*I*_ for the sake of illustration. The consistency process starts by selecting interest points from the image of *P* and the image of *R*_*I*_ to verify if their content matches. The method relies on scale-invariant features transform (SIFT) [31]. SIFT identifies interest points from the images and generates local descriptions from them (feature vectors) that are robust to scale, rotation, and color. The method stores the coordinates (x and y position within the image) and SIFT descriptions for each selected point. Using the interest point descriptions, the method matches the most similar interest points of *P* and *R*_*I*_ using a brute-force strategy (*i.e.*, checking all possible pairs of descriptions).

As expected, some matches from *P* to *R*_*I*_ are false. To consider only the consistent ones, the method finds a homography transformation *H* that aligns the largest possible number of matched points from *P* to *R*_*I*_. This transformation is depicted in the multiple green parallel lines of Fig 4 (step 2), which map *P* points into *R*_*I*_ points. Homography transformations will possibly re-scale, rotate, and translate the interest points of *P* to match its content to *R*_*I*_ [32]. To find the homography transformation and eliminate incorrectly matched interest points, we use the MAGSAC method [33].

As SIFT features are not robust to mirroring, we also perform the same interest point matching and image alignment in the mirrored version of *P*, named *P*′. If neither *P* nor *P*′ panels have at least 20 consistent matching interest points with *R*_*I*_, we consider the pair (*P*, *R*_*I*_) inconsistent and discard it.

#### 3.3.3 Content sharing score calculation

This step verifies if the content between *P* and *R*_*I*_ is relevant to an integrity analysis, taking into account the region of *P* that was matched with *R*_*I*_, as illustrated in Fig 4 (step 3). To delimit this region, the method finds the smallest convex polygon (a.k.a. convex hull) that involves all matched interest points from *P* to *R*_*I*_ (Fig 4, step 3). Then, using the area of this polygon divided by the total area of *P*, we calculate the *Content Sharing Score* of *P*. Mathematically, the content sharing score is given by the convex hull area built using the matched interest points from *P* to *R*_*I*_ divided by the total area of *P*. In the following equation, *P* ∩ *R*_*I*_ represents the *Content Sharing Score* of *P* with *R*_*I*_, while *A*(*x*) represents the area of *x*.

$$\begin{array}{c} P\cap R_{I}=\frac{A(P_{Convex\;Hull})}{A(P)} \end{array}$$ (1)

By comparing the content sharing score between *P* and *R*_*I*_ with a threshold, we can determine if the region of *P* matched to *R*_*I*_ is relevant for an integrity analysis. Conversely, if the content-sharing score is below the threshold, we consider the pair (*P*, *R*_*I*_) inconsistent and discard it.

The content sharing score is a value between 0.0 and 1.0, indicating the degree of similarity between the content of *P* and *R*_*I*_. A score close to 0.0 indicates that *P* shares few portions with *R*_*I*_, while a score close to 1.0 indicates that *P* shares most of its content with *R*_*I*_. It is important to note that the content sharing score of *P* ∩ *R*_*I*_ differs from that of *R*_*I*_ ∩ *P*, as these values are relative to each image. For instance, if *P* is a cropped version of *R*_*I*_ that covers 50% of the area of *R*_*I*_, then the content sharing score *P* ∩ *R*_*I*_ would be equal to 1.0, while the content sharing score *R*_*I*_ ∩ *P* would be 0.5.

#### 3.3.4 Content shared table building

In this stage, a table is generated to indicate the *Content Sharing Score* between *P* and all items in the processing queue (as shown in Fig 4, step 4). Using this table (interpreted as an adjacency matrix), the method determines the most relevant images for integrity analysis. After the content-sharing score of the pair (*P*, *R*_*I*_) is calculated, it is inserted directly into the table’s cell at row *P*, column *R*_*I*_. Similarly, the content-sharing score of the pair (*R*_*I*_, *P*) is placed in the cell at row *R*_*I*_, column *P*. An image is considered relevant for integrity analysis (*i.e.*, a possible duplication) if its content sharing score in the *Content Shared Table* exceeds a threshold. Empirically, a threshold of 1% is effective, meaning that the (*P*, *R*) pair is deemed suspicious if *P* ∩ *R*_*I*_ ≥ 0.01 or *R*_*I*_ ∩ *P* ≥ 0.01.

Note that if *R*_*I*_ is relevant to *P* (*i.e.*, they share content), other similar images to *R*_*I*_ may also be relevant to compare with *P*. Thus, the method expands the processing queue of *P* by adding the top-*L* most similar images of *R*_*I*_, similarly as performed in the *Content Retrieval* step, but using *R*_*I*_ as a query. We use *L* = 40 in our experiments. To avoid redundancy, images that have already been processed will not be included twice in the queue.

#### 3.3.5 Processing queue loop

Once panel *R*_*I*_ has been analyzed, it is removed from the processing queue, and the next panel is selected for examination. This process is repeated until either 300 panels have been analyzed or the queue becomes empty. Based on our experiments, panels with a queue position of 300 or higher generally have significantly different content from panel *P* and, therefore, do not provide meaningful evidence for the provenance analysis. While the processing queue is limited to 300 elements, it’s important to note that this queue only includes pairs of images that have not been processed previously, keeping in mind that each probe is processed in parallel. Thus, even if the number of retrieved items, K, is higher than 300, there may be a higher number of redundant pairs that have already been or are being analyzed in parallel, which will be ignored. For example, if P is the probe and *R*_*I*_ is within the top-*K* similarity ranking of P, when analyzing the top similar images to *R*_*I*_, there is a high probability that P will also be within the top-*K* ranking of *R*_*I*_. This explains why the top-*K* items retrieved are higher than the size of the processing queue and why including a second-tier retrieval rank in the processing queue increases the likelihood of finding new types of matches.

#### 3.3.6 Provenance graph construction

This step aims to group all items that share content and create a provenance graph visualization (as illustrated in Fig 4, steps 5 and 6). This visualization presents a set of images that relate to one another, tracking the image with the highest content-sharing score.

Before starting the *Graph Construction* step, all panels within the database should be processed through the previous steps, and their **Content Shared Table** must be filled. This allows the method to determine how much content an image shares with others. Subsequently, the method identifies and links all pairs of images with content-sharing scores greater than 1%, isolating them into groups. Within a group, each element is related to at least one other element in the same group. These groups are formally known as connected components in computer science graph theory.

**Fig 5:**
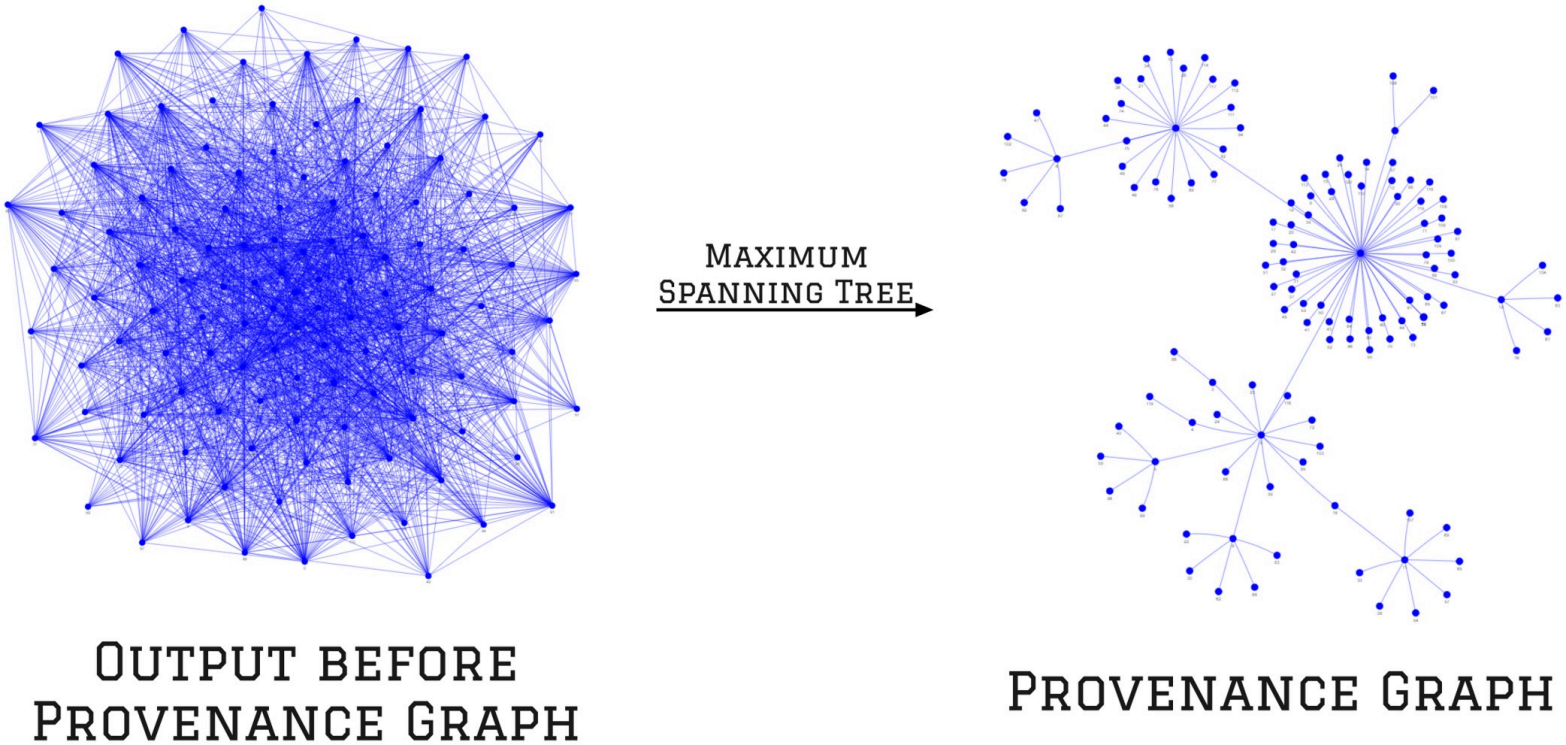
Visualization of a graph before and after computing its maximum spanning tree, referred to as the provenance graph. Each blue node represents a different image, and the links between nodes indicate that their corresponding images share content. On the left is the connected component graph, which shows a connected group of images identified by the proposed method. On the right, the corresponding provenance graph is obtained by pruning the links of the connected components graph by computing the maximum spanning tree (MST). MST removes all cycles within the graph while keeping the edges that maximize the sum of all content-sharing scores between each linked node.

Within a connected component, an image may be linked to multiple others, resulting in a dense graph that can be difficult to understand and visualize. Fig 5 illustrates an example of a connected component before and after improving its visualization. To enhance the visualization and identify duplicated images, the method removes all cycles within the connected components and preserves the links that maximize the sum of all content-sharing scores. Specifically, the method computes the maximum spanning tree, an undirected graph with the maximum weights on its links, for each connected component. Fig 5 shows the output before and after pruning the links of a connected component. The pruned version of the connected component is the provenance graph, which identifies images connected to numerous others, providing evidence of systematic production. For instance, the central blue node in Fig 5, which has the most connections to other nodes in the graph, may indicate the source of a systematic production and could be the starting point for a scientific integrity investigation.

### 3.4 Provenance analysis at document level

**Fig 6:**
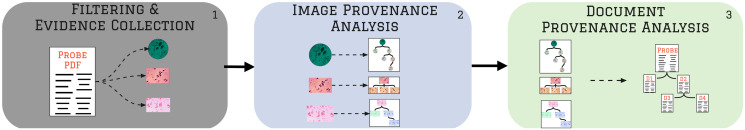
Provenance analysis at document level. The process of document-level provenance analysis begins with *Filtering & Evidence Collection* (1), followed by image-level analysis to identify relationships and graphs of the collected figures (2). Finally, it tracks related documents through their linked figures and creates a provenance visualization of them. The images used in this figure are public domain and were used only for illustration’s sake.

The goal of document provenance analysis is to indicate the group of documents that reuse elements from each other, providing clues of systematically produced articles. This analysis uses the content shared table from the image level to link the image source documents. Fig 6 depicts the complete investigation, namely 1. *Filtering & Evidence Collection*; 2. *Provenance Analysis at Image Level*; and 3. **Document Provenance Analysis**.

The document analysis starts by creating a squared table *M* with *n* rows and *n* columns, where *n* is the size of the document dataset. Each row and column in this table represents a document from the dataset, and the value of a cell *d*_*i*,*j*_ (row *i*, column *j*) represents the number of duplicated elements between document *i* and *j*. The content-shared tables from the image level are used to populate *M*. For instance, if document *D*_*i*_ shares *k* elements with document *D*_*j*_, the cell in row *i* (representing *D*_*i*_) and column *j* (representing *D*_*j*_) of *M* is assigned the value *k*. This table can be interpreted as the *adjacency matrix* of the documents. Next, the document analysis identifies the connected components of *M*, similar to the image level. To improve the visualization of these components, a document provenance graph is created (as illustrated in Fig 6, step 3). This graph represents the maximum number of shared elements between documents, similar to the graph constructed at the image level.

Through this analysis, we observed that articles produced by paper mills are connected in the same provenance graph, *despite having unrelated authors or subjects, i.e., we could find the needles from the haystack.*

## 4 Experimental settings

This section presents the experimental settings for evaluating the proposed solution. We organized the experimental settings in Sec. 4.1 Paper Mill Datasets, a data collection for testing paper mill detection techniques; Sec. 4.2 Baselines, state-of-the-art methods designed for a similar task as the proposed one; and Sec. 4.3 Evaluation Tasks and Metrics.

### 4.1 Paper mill datasets

Bik [11] and collaborators first reported a set of scientific articles suspected of belonging to paper mills. We started with their work and increased the amount and detail of the annotated problems. We also included distractor documents (*i.e.*, documents without known issues) to challenge any proposed solutions to work with a more realistic needle-in-the-haystack scenario.

#### 4.1.1 Stock Photos Paper mill (SPP) dataset

**Fig 7:**
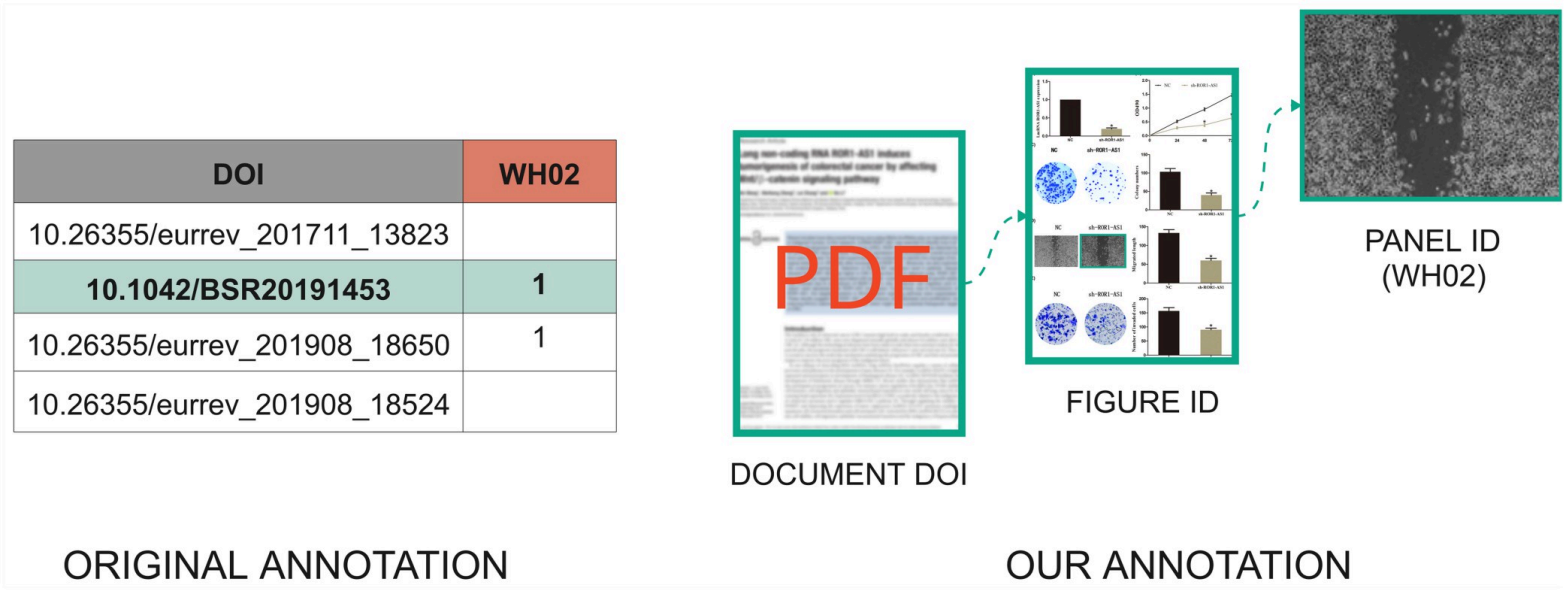
Extension of the SPP dataset annotations. The original annotations (left) rely on spreadsheets and offer limited information about the shared visual content. They indicate whether a document, identified by its DOI, reuses an image and which label corresponds to the reused image. In this instance, “WH02” represents a label for a group of similar wound-healing assay photos identified across multiple articles. The original spreadsheet annotation and its detailed explanation are described on Dr. Bik’s website [11]. The proposed new annotations (right) rely on documents in JSON format to track and register all the figures within a document and all the panels within a figure that suspiciously share regions. The panel in this figure is present in the dataset and extracted from https://doi.org/10.1042/BSR20191453 under a Creative Commons license.

The SPP dataset contains 121 biomedical articles describing cancer types and cell tissue samples. Bik [11] and collaborators annotated the suspicious occurrences of similar images throughout these papers. Such annotations were made publicly available on Bik’s website [11] via spreadsheets. As one might observe on the leftmost panel within Fig 7 (see “Original Annotation”), each row of a spreadsheet represents a particular article; it contains a column to identify the publication through its Digital Object Identifier (DOI), and a column with its *label*.

As a limitation of this annotation, one cannot pinpoint the similarities and shared visual content between two papers with the same label. To provide more complete annotations with a focus on media forensics, we are now detailing the content-sharing relationships between pairs of papers at the level of *documents* (121 samples), *figures* (498 samples), and *panels* (2,581 items). The latter level (panels) is grouped into categories (*e.g.*, panels that depict Microscopy Imagery, Western blots, Graphs, etc.). Fig 7 puts in perspective the original SPP dataset spreadsheet-based annotations (on the left side) with the herein proposed ones (on the right side). The new annotations are stored in JavaScript Object Notation (JSON) [34] to cope with the more complex and more complete information.

#### 4.1.2 Extended SPP dataset versions

Aiming to challenge the proposed solutions and understand their performance regarding false alarms (when programs wrongly accuse issues over non-problematic data), we extended the SPP dataset by adding documents without known problems (distractors). To make more realistic scenarios –where the majority of the analyzed papers do not have problems– and to understand the progression of the challenge over large and larger-sized corpora of articles, we created two versions of the SPP extension. In the first one (namely “v1”), 969 papers containing biomedical figures were added to the SPP dataset. Each paper was found through its figures, which were queried using the Open Access Biological Image Search Engine [28]. We explicitly selected interest figures via the engine’s categories, such as Microscopy images, Flow Cytometry, and Western blots. In the second version (namely “v2”), 3, 635 papers were similarly added to the first version. All added articles were not associated with any suspected image-related misconduct.

Table 2 summarizes the respective numbers of the annotated documents, figures, and panels that constitute the SPP, extended SPP (v1), and extended SPP (v2) datasets. Table 3 presents the distribution of image panels by type for each version of the SPP dataset. The original SPP annotation did not include Body Imaging panels. Still, given their high prevalence of image reuse, we added them to the extended versions of the SPP to serve as distractors.

**Table 2:** Number of items per SPP dataset.

Dataset	Documents	Figures	Panels
**SPP**	121	498	2,583
**Extended SPP (v1)**	1,090	1467	10,145
**Extended SPP (v2)**	4,725	5,303	47,540

Extended versions “v1” and “v2” aim to increase the challenge posed to the proposed solutions and represent more realistic scenarios where most analyzed papers do not present problems.

**Table 3:** Distribution of image panel types across the SPP datasets.

Panel Type	SPP	Extended SPP (v1)	Extended SPP (v2)
**Microscopy**	925	4,227	14,083
**Blots**	278	1,298	9,810
**Body Imaging**	0	573	10,715
**Graphs and Plots**	1,317	3,620	9,879
**Flow Cytometry**	63	427	3,053
**Total**	2,583	10,145	47,540

Although Graphs and Plots panels were included in all versions of the dataset, we did not consider such a category during our experiments. This category involves figures generated analytically from tabular data. Due to their nature and visual appearance, they often look very similar despite coming from different data, leading to many false connections when visually analyzing such figures. Therefore, we do not recommend applying our provenance analysis method to these images; instead, we recommend doing statistical analysis, which is out of the scope of this work. We opt to keep this category in the datasets to facilitate future work with a handy dataset for such analysis.

### 4.2 Baselines

We selected two state-of-the-art methods previously proposed in the literature of digital forensics and scientific integrity as baselines to put the herein-proposed solution into perspective:

- **Bioscience-scale automated detection of figure element reuse (BSRD).** Acuna *et al.* [17] introduced a human-assisted methodology to detect image reuse in biomedical databases. Their pipeline contains one module for copy-move detection and another for biological image classification. The copy-move stage is an interest point-based extraction and matching algorithm similar to the one developed by Amerini *et al.* [35], except for an adaptation to the case of scientific images (*e.g.*, microscopy imagery) instead of natural scenes (*e.g.*, outdoor landscapes).
  We were unable to locate an open-source implementation of the copy-move detection method developed by Acuna *et al*. Therefore, we developed our own version based on their article, which we employed in our experiments. As Acuna *et al.* solution does not provide a provenance outcome, to effectively compare our solution with Acuna *et al.*, we recorded the number of matching points identified by their copy-move detector for each input image pair in an adjacency matrix (the content-sharing table). During that process, we carefully considered any constraints applied by their method, such as discarding matched pairs with three or fewer matching points. Our in-house implementation is available on the GitHub repository of this article. Throughout the article, we refer to our implementation of Acuna *et al.*’s method as **BSRD**, which stands for Bioscience-Scale Reuse Detector.
- **SILA.** A System for Scientific Image Analysis (*SILA*) [18], which contains several modules (including a provenance analysis one) from the literature of digital forensics tuned to scientific images. SILA’s provenance analysis is based on the work of Moreira *et al.* [19] and Bharati *et al.* [20], with components added to cope with scientific images (*e.g.*, a text detection component that avoids matching sub-panel legend letters within the scientific figures). During the experiments, we used the available implementation of SILA official GitHub repository (https://github.com/danielmoreira/sciint/tree/master).
  In contrast to the proposed solution herein, *SILA*’s method was designed to find the relationships of a single query image with a scientific image corpus. On the other hand, our solution is designed to find **all relationships within a collection of either document (document level) or their inner figures (image-level)**, with no need for an explicit query image of interest. Additionally, our method does not operate on the entire scientific figure. This deliberate choice eliminates the need for OCR systems and mitigates the issue of text matching. However, for a fair comparison, we used the panels extracted by our solution as input to the SILA method during the experiments.

### 4.3 Evaluation tasks and metrics

To verify the effectiveness of the proposed solutions and baselines from the literature, we assess their performance in the face of three tasks:

- Finding images and documents that share content one with another (Content Pairing).
- Grouping and determining the categories of reused elements within a collection of scientific images and documents (Content Grouping).
- Classifying images and documents as either produced by a paper mill or not (Content Classification).

Results are organized into two levels: image versus document. The first one is dedicated to finding forensic traces that link the scientific images (*i.e.*, analysis at image level), while the second one aims at finding shared content between the documents (*i.e.*, analysis at document level).

#### 4.3.1 Content Pairing (CP)

This task assesses the effectiveness of a method in identifying image pairs with shared content or publication pairs that reuse the same images. Thus, if an image *I*_*A*_ has any portion of its content reused by another image *I*_*B*_, we consider that *I*_*A*_ is linked to *I*_*B*_. Similarly, if a document *D*_*A*_ shares elements from another document *D*_*B*_, then *D*_*A*_ is linked to *D*_*B*_. To assess the capability of a solution to find these links, we rely on the metrics link precision and link recall. Link precision (*LP*) denotes the number of relationships correctly found by a solution divided by the total number of links returned by the solution. It thus aims to answer the following question: “From the links computed by the solution, how many were predicted correctly?” Link recall (*LR*), in turn, denotes the number of relationships correctly found by a solution divided by the total number of links annotated in the dataset. It answers the following question: “From the links annotated in the dataset, how many were correctly found by the solution?” As expected, we want *LP* and *LR* close to 1.0 (best result). Ultimately, we set *CP* as the harmonic mean of *LP* and *LR* (see Eq 2). This metric is inspired by the Edge Overlap (*EO*) concept proposed by NIST [36]. The closer to 1.0 the value of *CP*, the better the solution.

$$\begin{array}{c} CP=\frac{2\times LP\times LR}{LP+LR} \end{array}$$ (2)

#### 4.3.2 Content Grouping (CG)

This task measures the effectiveness of a method in identifying and grouping images that share content (image level) or publications that reuse the same images (document level). This task was inspired by the image categories identified by Dr. Bik and other investigators [11], such as the colony formation assay photo category one (*CF1*), which includes multiple similar images reused across various articles.

To assess the effectiveness of solutions for this task, we use node precision (*NP*) and node recall (*NR*). *NP* is the number of correctly identified images or documents (nodes) included in the predicted group divided by the number of elements annotated for this group. *NP* answers: “How many items in a predicted group correctly belong to the same category?”. On the other hand, *NR* is the number of elements from the same category correctly grouped, divided by the annotated number of elements in that category. *NR* answers: “From an annotated category, how many elements did the solution correctly identify and group?”. As close *NR* and *NP* are to 1.0, the better the solution. Similarly to the case of *CP*, *CG* uses the harmonic mean of *NP* and *NR* (see Eq 3). *CG* is inspired by the metric Node Overlap (*NO*) concept proposed by NIST [36]. *CG* value is within the interval [0, 1]. The closer its value to 1.0, the better the solution.

$$\begin{array}{c} CG=\frac{2\times NP\times NR}{NP+NR} \end{array}$$ (3)

#### 4.3.3 Content Classification (CC)

This task determines whether a picture or publication is systematically produced (*i.e.*, created by a paper mill). It answers: “How effective is a solution in identifying suspected items from paper mills?”. In the dataset, reused images (image level) and articles (document level) reported by Dr. Bik and other investigators were marked as suspicious, while all other items were considered not suspicious.

To assess *CC*, we rely on *precision* and *recall* metrics. Precision is the ratio of correctly identified suspicious items to the total number of items identified by the solution. Recall is the ratio of elements correctly predicted as suspicious to the total number of suspicious items added to the dataset.

To address the imbalance in the SPP extended datasets (*i.e.*, the number of suspicious elements lower than unsuspicious), CC uses the harmonic mean of precision and recall (Eq 4). CC ranges from 0.0 to 1.0, with higher values indicating better performance.

$$\begin{array}{c} CC=\frac{2\times precision\times recall}{precision+recall} \end{array}$$ (4)

An important aspect of the three evaluation tasks (*i.e.,* CP, CG, and CC) is that they are designed to work in tandem, providing a comprehensive assessment of a solution. While content classification focuses on identifying problematic content, it cannot evaluate whether the links identified by a solution actually connect two problematic items. For instance, a solution might link a problematic image to a pristine one, yet the problematic image would still be correctly flagged in the content classification task. In turn, the content grouping task offers complementary insights to content pairing by leveraging the quality of the links assessed during content pairing to effectively group elements that share the same content. In contrast, content pairing provides an evaluation of the links, specifically assessing how accurately a solution identifies reused content.

## 5 Results

We organized the outcomes of this work in Sec. 5.1 Quantitative Results, including effectiveness analysis for image reuse and systematic production detection; Sec. 5.2 Qualitative Results with analysis and output visualization of the proposed method herein; and Sec. 5.3 Automated Panel Extraction performance analysis.

### 5.1 Quantitative results

We present results at both the image and document levels for each evaluated task. During the quantitative analysis, we excluded Graphs and Plots image panels, as their evaluation requires statistical analysis.

Additionally, we conducted an analysis by image type, assessing the performance of each method individually for Microscopy, Blots, and Flow Cytometry panels. Body Imaging panels were not included in the individual analysis because they are absent from the original SSP dataset and were only added as distractor panels. Consequently, there is no annotated connection among Body Imaging panels.

#### 5.1.1 Content Pairing (CP)

Table 4 reports the *CP* results at the image level for each version of the SPP dataset. As the number of distractors increases from SPP to Extended SPP (v1) and Extended SPP (v2), *BSRD* and *SILA* performance decrease due to the rise of false positive links computed by these methods. In turn, the proposed solution is effective over all dataset versions, varying down only three percentage points in the worst scenario of the Extended SPP (v2), which presents more distractors.

**Table 4:** Content pairing results at image level.

Dataset\Method	BSRD	SILA	Proposed
**SPP**	0.54	0.72	**0.74**
**Extended SPP (v1)**	0.25	0.66	**0.74**
**Extended SPP (v2)**	0.04	0.36	**0.71**

The best results are in bold. The proposed solution is more robust to the addition of distractor images than the others.

**Fig 8:**
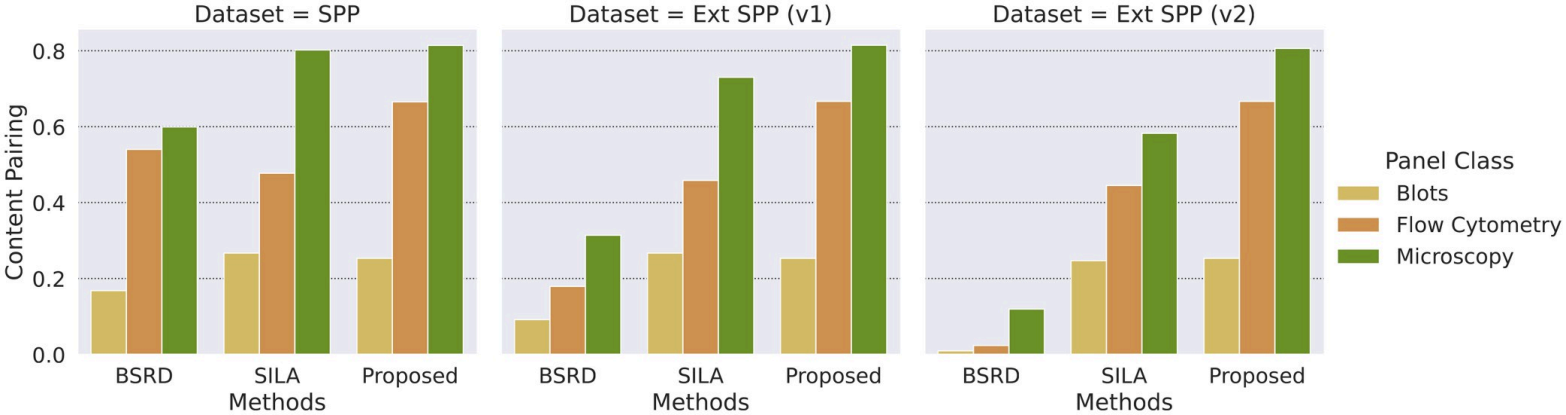
Content pairing results per image type for each SPP dataset version. Blots are the most challenging type of image for all solutions. Contrary to the other solutions and regardless of the version of the SPP dataset, the proposed method does not suffer significant drops of *CP* in the presence of distractors.

Fig 8 presents the values of *CP* obtained by each solution over the three versions of the SPP dataset. *CP* values are grouped according to the type of image (blots, flow cytometry, and microscopy imagery). The proposed solution consistently maintains its capability of linking reused images across the different versions of the dataset, indicating stronger robustness to the presence of distractors. Blots present the most challenging image type, with low scores for all solutions. They are typically shown in low-entropy images with undefined boundaries and blurred backgrounds (see Fig 3, yellow rectangles). These characteristics complicate the extraction of keypoints, which are crucial for image matching in all solutions, likely contributing to the low performance observed for this type of image and the difference in performance for the other image types.

Table 5 reports the results of *CP* at the document level. The slight difference in performance between SILA and the proposed method indicates that both solutions perform similarly for this task and are stable in detecting reused images across different documents, even in scenarios with multiple distractors. (*i.e.*, documents without known image reuse issues).

**Table 5:** Content pairing results at document level.

Dataset\Method	BSRD	SILA	Proposed
**SPP**	0.72	**0.84**	**0.84**
**Extended SPP (v1)**	0.28	**0.84**	0.83
**Extended SPP (v2)**	0.03	0.73	**0.77**

The best results are in bold. The proposed solution works on par with *SILA*, presenting a better *CP* in the extended SPP (v2), which is the most challenging one.

On the other hand, BSRD (our implementation of Acuna *et al.*’s method) significantly drops its performance in the scenario with distractors.

#### 5.1.2 Content Grouping (CG)

Table 6 expresses the *CG* results at the image level. In contrast to *BSRD* and *SILA*, the proposed solution exhibits only a slight deviation across all dataset versions, with a difference of only four percentage points in the dataset version with the most distractors.

**Table 6:** Content grouping results at the image level.

Dataset\Method	BSRD	SILA	Proposed
**SPP**	0.81	0.81	**0.84**
**Extended SPP (v1)**	0.24	0.74	**0.84**
**Extended SPP (v2)**	0.02	0.42	**0.80**

The best results are in bold. Contrary to the other solutions, the proposed one is slightly affected by the presence of distractors.

**Fig 9:**
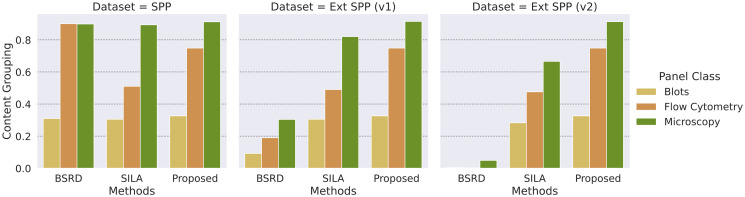
Content grouping results per image type for each SPP dataset version. Blots are the most challenging type of image for all solutions. Like the case of *CP*, the proposed method does not suffer significant drops of *CG* in the presence of distractors.

Fig 9 depicts the *CG* performance of the evaluated method. Similarly, as in the case of *CP* results, Blots also pose the most challenging type of image.

At the document level, *CG* considers the group of documents that belong to the same paper mill. Given that we only included one group of suspect papers, the SPP, we expected all 121 publications to be included in the same group. Table 7 reports the CG results at the document level. As expected, considering only the articles from SPP (no distractors), all solutions scored close to 1.0, the maximum score. However, when approaching real-world conditions, by including more and more distractors from Extended SPP (v1) to Extended SPP (v2), all solutions lose effectiveness. *BSRD* solution is the most affected dropping the *CG* value from 1.00 to 0.002 (rounded to 0.00 in Table 7). The proposed solution has the lowest impact when facing distractors, dropping only eight percentage points when facing the worst scenario. This experiment indicated that when dealing with a more realistic and complex scenario (using the extended SPP datasets), most methods created false-positive links between previously established groups and the distractors.

**Table 7:** Content grouping results at document level.

Dataset\Method	BSRD	SILA	Proposed
**SPP**	**1.00**	0.99	**1.00**
**Extended SPP (v1)**	0.15	0.99	**1.00**
**Extended SPP (v2)**	0.00*	0.86	**0.92**

*BSRD scored 0.002, which was rounded to 0.00.

The best results are in bold. As the number of distractors increases across the evaluated datasets, the performance of all methods decreases. However, the proposed exhibited the most robust performance across different numbers of distractors.

#### 5.1.3 Content Classification (CC)

Table 8 indicates the *CC* results at the image level for each SPP dataset version. As expected, when applied to the isolated images of SPP, all solutions have a high performance. However, with the addition of image distractors, *BSRD* and *SILA* failed to identify the suspicious images. In turn, the proposed method kept its high performance in all scenarios, indicating robustness to distractors.

**Table 8:** Content classification results at the image level.

Dataset\Method	BSRD	SILA	Proposed
**SPP**	**0.87**	0.84	**0.87**
**Extended SPP (v1)**	0.44	0.77	**0.87**
**Extended SPP (v2)**	0.08	0.44	**0.84**

The best results are in bold. The proposed solution outperforms the other evaluated techniques on all datasets for the task of content classification.

**Fig 10:**
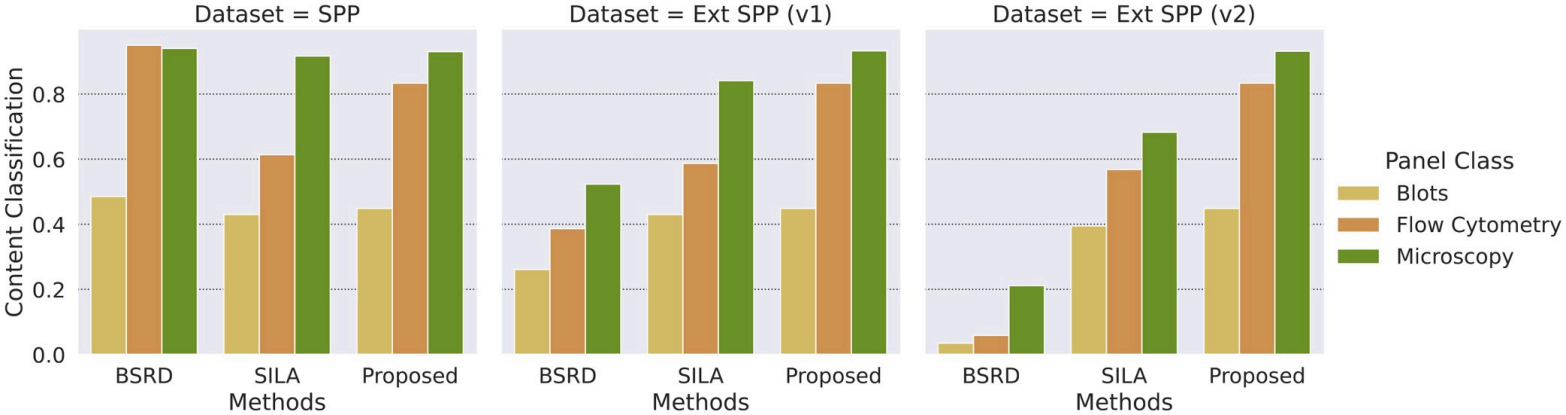
Content classification results per image type for each SPP dataset version. Unlike the other solutions, the proposed method does not suffer significant drops of *CC* in the face of unsuspicious data.

Fig 10 depicts the *CC* performance of each evaluated method. Similar to all other tasks, the results indicate that identifying Blots poses the biggest challenge for *CC*.

Table 9 reports the *CC* results at the document level. As expected, all solutions achieve *CC* values close to 1.0 when applied to the dataset without distractors. However, when the number of distractors is increased, the performance of the solutions diminishes. Specifically, when non-problematic items are added to the dataset, *BSRD* solution is the most affected, with a drop of 96 percentage points in *CC*. In contrast, the proposed solution is robust when the haystack size increases, maintaining stable and higher *CC* values across all dataset versions.

**Table 9:** Content classification results at the document level.

Dataset\Method	BSRD	SILA	Proposed
**SPP**	**1.00**	0.99	**1.00**
**Extended SPP (v1)**	0.44	0.99	**1.00**
**Extended SPP (v2)**	0.07	0.86	**0.92**

### 5.2 Qualitative results

In this section, we present the provenance graphs generated by our solution when applied to the SPP dataset (v2), providing an explainable and user-friendly visualization of the images and documents identified as being produced by paper mills. Furthermore, the qualitative results are presented separately for image level and document level, providing insights into the performance of our solution in each context.

#### 5.2.1 Image level

Our solution provides a provenance graph visualization in which the graph nodes represent images and the links represent their relationships of reuse. For example, a link from a node *A* to another node *B* indicates that part of *A* is reused in *B*.

**Fig 11:**
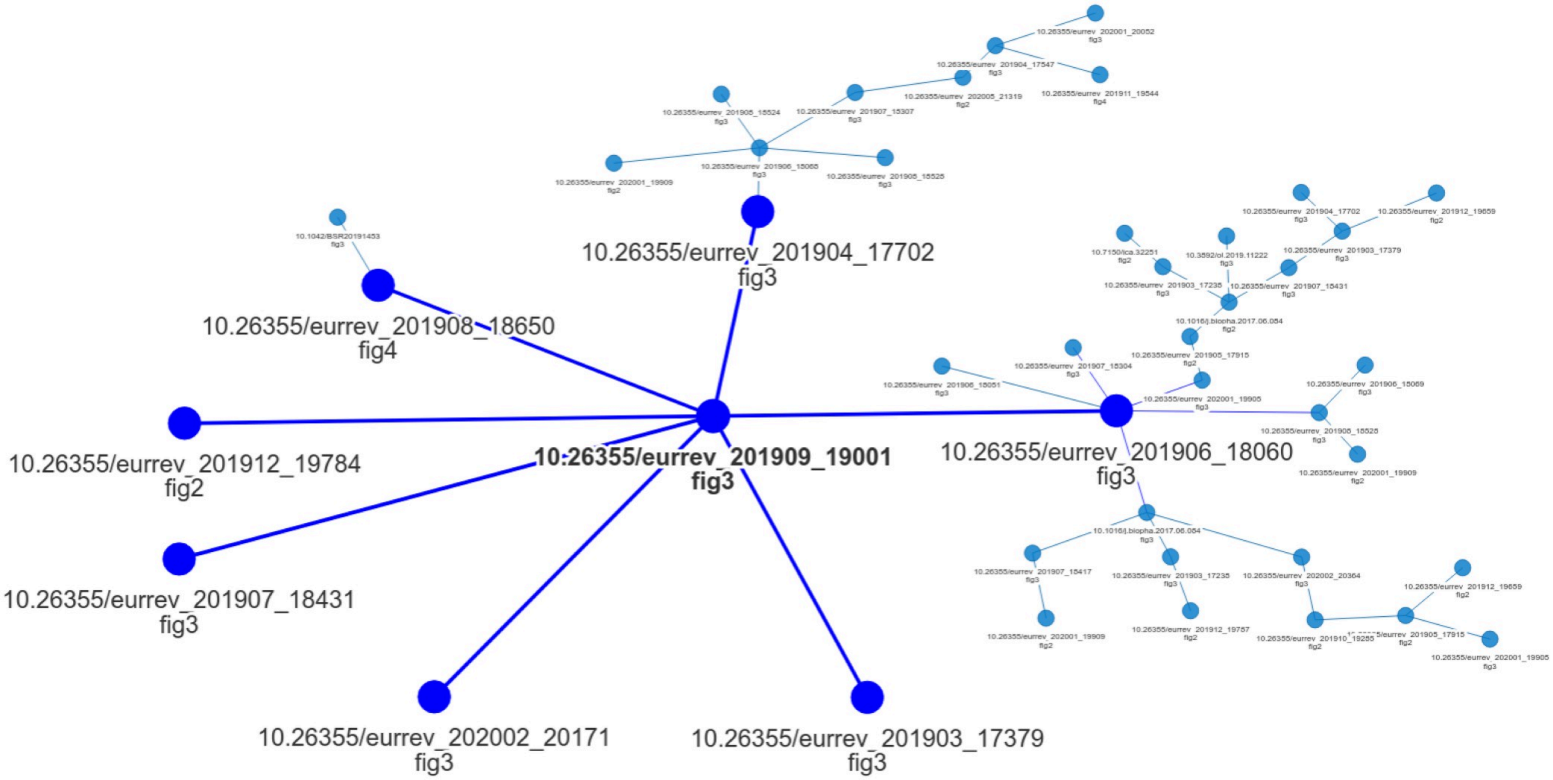
Provenance graph computed by the proposed solution herein over the extended SPP dataset (v2). Each graph node refers to an image panel from a scientific figure reused in a document. Blues lines linking each pair of nodes indicate the sharing of visual content. To improve visualization, we select one node from the graph and highlight its neighbors, increasing the size of the nodes and coloring them with a stronger blue color. We did not include the source figures in the graph due to copyright issues. Below each node, we provide the document object identification (DOI) of the source manuscript of the involved figure and the reference used in the article to such figure.

Fig 11 depicts the provenance graph generated by our solution when applied to the SPP Extended (v2) dataset. Each node in the graph represents an image labeled with its digital object identification (DOI) and a reference to the reused figure. This graph has the largest number of reused images among all the output graphs and consists entirely of microscopy images. After comparing Fig 11 graph with Bik’s annotations [11], we have found that the automatically-computed outcome matches the category labeled as “TW14” within their annotation schema. **All “TW14” images were correctly found** and matched by our method, not including any false positive link.

**Fig 12:**
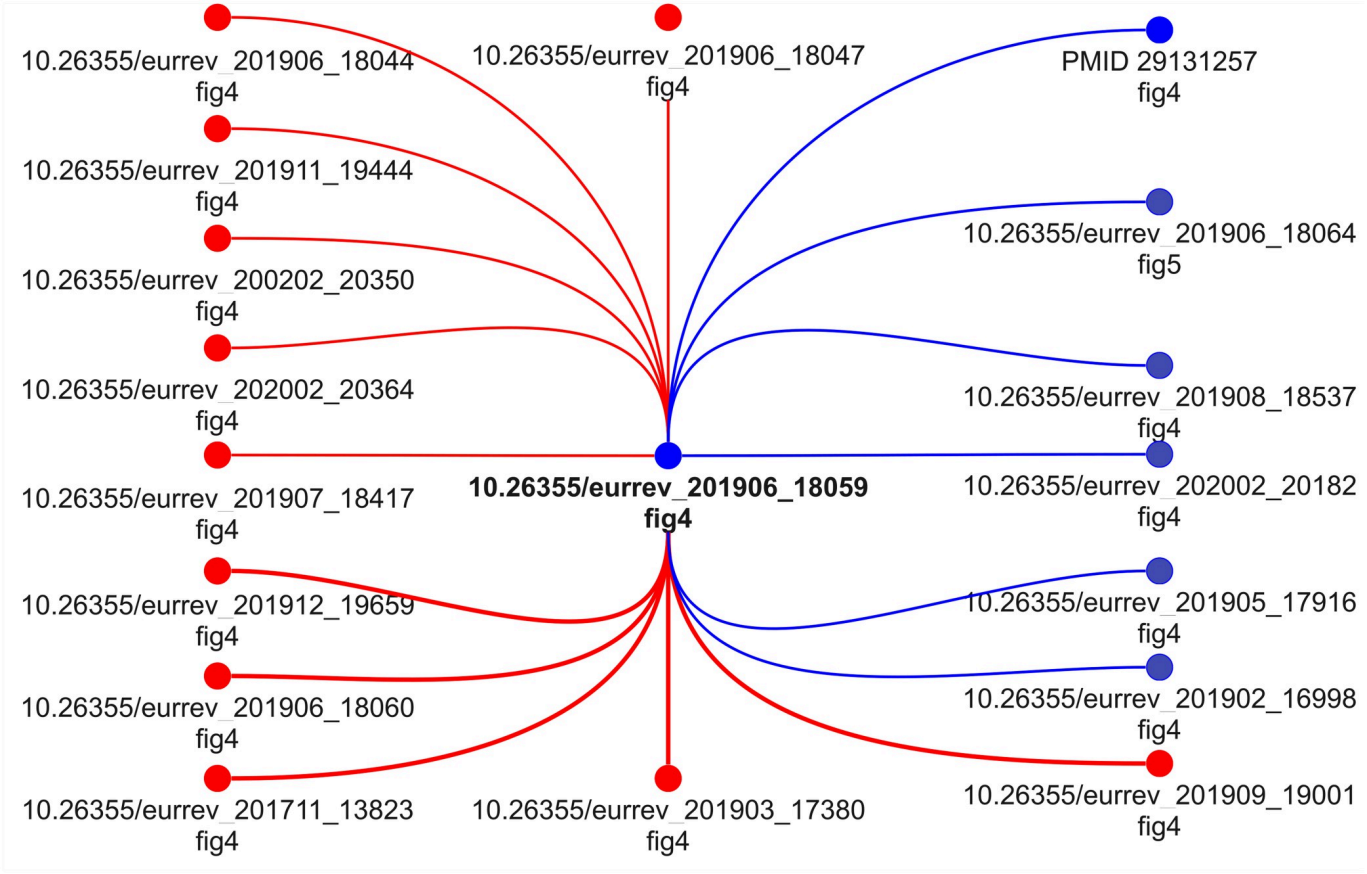
Provenance graph of Western blots related to the group labeled as “SWB1” within Bik’s annotations [11]. Blue nodes refer to correctly predicted figures. Red nodes indicate missed figures not found by the proposed method. Due to copyright issues, we did not include the real figures in the graph. Below each node, we provide the DOI of the document that is the source of the involved figure, as well as the figure’s reference used in the document.

Fig 12 depicts the output of our method when applied to Western blots. Blue nodes represent correctly found figures from the image group labeled as “SWB1” within Bik’s annotations [11]. In turn, red nodes comprise the images our method failed to detect (*a.k.a.* misses or false negatives). As presented throughout the quantitative results for all the tested methods, Western blots are a challenging type of image to find regions of content sharing. The graph presented in Fig 12 shows that only seven out of eighteen images within the group “SWB1” were correctly predicted as sharing content. This result indicates both a limitation of our method and a research opportunity for future work.

#### 5.2.2 Document level

**Fig 13:**
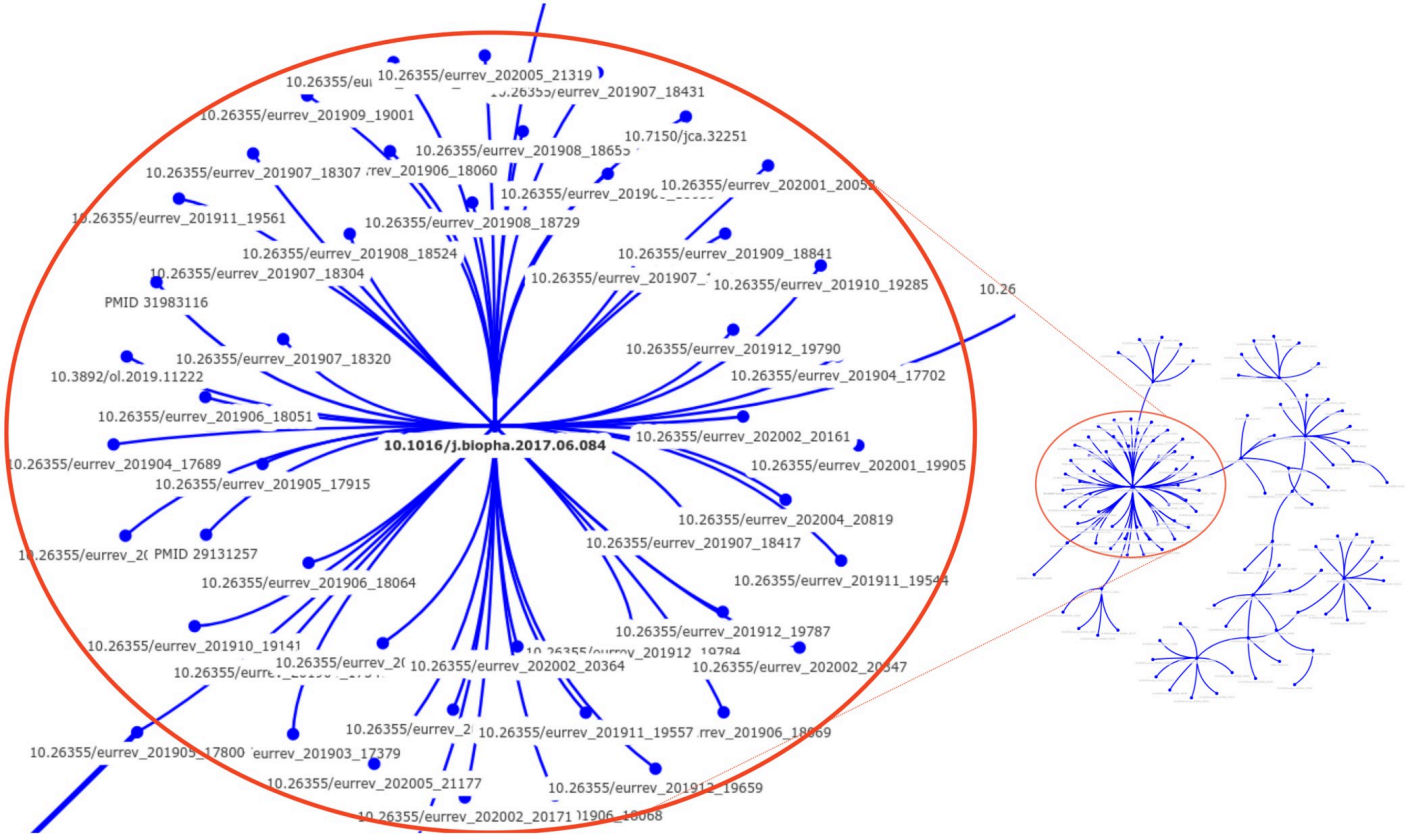
Provenance graph generated by the proposed solution, representing the document level relationships between articles sharing content in the extended SPP dataset (v2). All documents within this graph were reported as problematic by Dr. Bik’s investigation [11], without false positives. Each node in the graph corresponds to a publication, with its DOI indicated below. The most densely connected region of the graph is magnified, revealing a document that shares its content with many others.

Fig 13 illustrates the largest graph generated by the proposed solution when applied to the extended SPP dataset (v2). Each node in the graph represents a scientific document that shares content with another. Upon comparing this graph with the paper mill documents identified in Dr. Bik’s investigation [11], we found that **all suspect publications were correctly included** without adding distractor articles.

**Fig 14:**
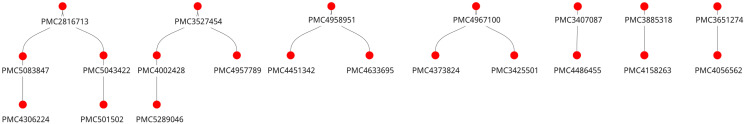
The seven false alarm provenance graphs detected by the proposed solution when applied to the SPP-v2 dataset. The red nodes represent publications identified by their PubMed Central (PMC) ID, displayed below each red node. Connections between nodes indicate potential image duplication between their articles detected by our solution. When reviewing the cause of the connection, we have found similar images in all connected articles, but most properly citing their sources. All flagged publications refer to distractor documents added to the extended SPP dataset, indicating false connections. Out of 4096 distractor documents included in the SPP-v2 dataset, only 21 were flagged as false alarms.

Fig 14 contains additional provenance graphs predicted by the proposed method over the extended SPP dataset (v2). Below each graph node (which represents a publication), there is a PubMed Central identifier (PMC); it refers to one of the distractors we have added to extend the SPP dataset and, therefore, comprises false alarms (only 21 out of 4604). Differently from the previous graph containing all reported articles, these additional seven graphs have smaller sizes, with at most five nodes. To understand the reason for such cases, we analyzed the images identified as false alarms by the method. We found that there is a high visual similarity among these images. Notably, most detected images are creative commons material commonly used as illustrations in the biomedical field. Additionally, these images were properly cited during their reuse and should not raise any concerns for human reviewers. For example, the rightmost graph links the document PMC3651274 with PMC4056562 after our method found Fig 1 of PMC3651274 to be very similar to Fig 2 of PMC4056562. Both figures were reused with proper citations and are not problematic. These false positives underscore the need for expert analysts to review the method’s output, given that genuine research can reuse images from other works ethically.

### 5.3 Automated panel extraction

Panel extraction is essential to focus on the image regions (*i.e.*, panels) of interest to the scientific integrity problem and filter out those that might raise false alarms due to their intrinsic similarity (*e.g.,* diagrams, drawings, and legend indicative letters). For this purpose, we collected and annotated 3,836 biomedical scientific figures under Creative Commons license from different journals, creating a dataset of 3,236 figures (32,507 panels) for training the extractor and 600 figures (4,888 panels) for testing it. Each figure’s URL and annotations are available in the GitHub repository of this work.

The proposed panel extractor is based on Yolo-v5 [25], an object detection model for digital images. Given our biomedical figures scope, we trained the solution to pinpoint the location of the most problematic biomedical types of panels [4, 14, 24], namely *Microscopy Images*, *Blots*, *Body Imaging*, *Graphs and Plots*, and *Flow Cytometry*.

We evaluate the proposed technique using the average precision (AP), a popular metric in computer vision for object detection [37]. The higher the AP, the more accurate the detection. Typically, AP is used with a parameter (*i.e.*, a threshold) that will only consider a panel as successfully detected if its overlap (*i.e.*, intersection over union, namely IoU) with the annotated area is higher than the threshold. In this study, we considered an IoU threshold of 0.5 (AP @ 0.5). The model achieved an AP score of 93.4% on the test set. Table 10 shows the model’s performance on the test set per type of panel. The herein-proposed panel extractor achieved high scores for all evaluated classes.

**Table 10:** Performance of the panel extraction solution by image panel type.

Panel Type	AP @ 0.5
**Blots**	0.996
**Graphs**	0.958
**Microscopy**	0.922
**Body Imaging**	0.825
**Flow Cytometry**	0.969
**Mean AP @ 0.5**	**0.934**

We report the average precision with an intersection over union of at least 0.5 (AP @ 0.5). A perfect solution would lead to an AP value of 1.0.

## 6 Conclusion

Given the growing concerns regarding paper mills, this research sought a possible solution for unveiling paper mill productions in large datasets. To achieve this, our proposed method leveraged provenance analysis to identify questionable documents within a suspect collection. After analyzing reported paper mill cases spanning the years from 2010 to 2024, we noted a significant concentration of suspected paper mill activities within the biomedical domain. Given this observation, we designed our solution specifically to address the challenges within this field, which might require adaptation to other domains.

After applying our proposed filtering stage, our experiments showed that all evaluated solutions were effective when applied to small document sets containing only paper mill-related articles, as indicated by all evaluated metrics. However, when applied to larger datasets with multiple distracting articles (*i.e.*, non-problematic papers), only the proposed solution remained stable with a high effectiveness score, varying only a few percentage points for all metrics. Thus, in contrast to the baselines, the proposed method proved more robust in a realistic scenario where most analyzed papers do not have problems.

The most challenging task we evaluated was *Content Pairing*. Our proposed solution scored 71% (the score value ranges between 0 and 100%) when applied to the *Extended SPP (v2)* dataset. Our method scored higher than the others, as Moreira *et al.* [18] scored 36% and Acuna *et al.* [17] scored 4%, but there is still room for improvement. These improvements could be mainly concentrated on Western blot images, which were particularly challenging and obtained the lowest scores for all evaluated solutions, indicating a future research opportunity.

When comparing the performance of all methods across different image types, we observe a trend: the methods perform best on Microscopy images, followed by Flow Cytometry, with the worst performance on Western blots. A possible explanation is that Microscopy images often contain more distinct elements, making them easier to match than Flow Cytometry or Western blots. Flow Cytometry images, characterized by large uniform areas (often white), pose challenges for content matching. In turn, Western blot images often have low entropy (*i.e.*, their foreground objects do not have a clear boundary), making it difficult for SIFT-based methods—used in all evaluated solutions—to find the best points for matching. Future research could focus on developing more advanced techniques to improve matching for each type of image.

Also, the visualization of provenance graphs presents a notable challenge as these graphs can become increasingly complex with a higher number of links and nodes. In our proposed method, we addressed this challenge by calculating the maximum spanning tree of the output graph, which helps reduce clutter and enhance visualization. This approach prioritizes links between nodes that share larger areas while eliminating links that create cycles and clutter.

While we have been satisfied with the results from the maximum spanning tree, it is important to acknowledge the potential for future research to explore alternative visualization methods capable of preserving all graph links while maintaining clarity and intelligibility. For instance, phylogenetic tree visualization techniques [38] could be explored, as these trees are analogous to provenance graphs. Additionally, another research opportunity delves into exploring other properties of the figures, such as their metadata or their caption, to improve visualization and unveil the links of suspect articles. That could potentially yield valuable insights for integrity analysts when analyzing other aspects of the paper mill content.

Overall, our results indicate that this work provides a viable solution using provenance analysis for the recently reported paper mill cases from the biomedical area. Nonetheless, extending this investigation to other scientific domains is essential, as they might require fine-tuned parameters. Furthermore, the future of paper mills remains uncertain, and further research and discussions are necessary to address such a challenging problem in all fields of science. Eventually, paper mills will adopt artificial intelligence algorithms to generate realistic synthetic scientific figures. Although this may seem like a futuristic threat, recent studies have demonstrated the ability of Generative Adversarial Networks (GANs), a type of deep learning method also used for creating *deepfakes*, to create convincing Western blots even on low computational power devices such as simple laptops. Investigating the potential of such a deep learning algorithm, Qi *et al.* [39] were able to artificially create realistic Western blots that even biomedical specialists were unable to distinguish from pristine ones.

Because of these new threats and the ones that may appear, our endeavor to develop integrity tools alone may not be sufficient to assure scientific integrity. We believe such a complex problem should be addressed by a multi-pronged approach that leverages technology, policy, and education to create a culture of integrity and accountability within the scientific community to prevent scientific misconduct, such as paper mills.

## Supporting information

S1 Appendix S1 Appendix includes additional details about the automated panel extractor solution and a study supporting the choice of each parameter in the proposed provenance workflow. (PDF)
