# Supplementary material for: Unveiling scientific articles from paper mills with provenance analysis
Source: PLoS One. 2024 Oct 30;19(10):e0312666. doi: 10.1371/journal.pone.0312666 (PMC11524478; doi:10.1371/journal.pone.0312666)
Supplement: S1 Appendix — (PDF) [file pone.0312666.s001.pdf]

S1 Supporting Information

This supporting information provides additional details about the automated panel extractor and an investigation of the parameters used in the provenance analysis workflow.

Automated Panel Extraction Training Details

In this section, we detail the training setup of the proposed panel extraction solution.

Panel extraction dataset

The proposed panel extraction dataset comprises 3,836 scientific figures collected from 563 different publishers. Most of these figures come from open-access articles, with the top-five sources being *Plos One, Scientific Reports, Oncotarget, Journal of Medical Case Reports, and BMC Cancer*. Since each journal has different guidelines and requirements for scientific images, the resulting layout of the panels within these figures can also vary. Therefore, when creating the test set, we aimed to maintain the most diverse distribution possible.

To achieve this, we selected all publishers with at least three scientific figures from our collection, resulting in 200 publishers. From these, we randomly selected three figures from each publisher, creating a test set of 600 figures. The remaining figures were included in the training set. This approach aimed to ensure a diverse and representative test set, reflecting a wide range of publisher guidelines and image layouts.

In summary, the panel extraction test set comprises 600 figures (three for each different publisher), of which we annotated 4,888 panels, and the train set contains 3,236 compound figures with 32,507 annotated panels. Table S1 presents the image panel distribution for each set.

Table S1. Distribution of panel images in the panel extraction dataset per category

Category	Train	Test
**Microscopy**	8,018	1,838
**Blots**	7,708	804
**Body Imaging**	9,763	379
**Graphs and Plots**	4,641	1,618
**Flow Cytometry**	2,377	249
**Total**	32,507	4,888

Panel extraction details

The proposed panel extraction solution fine-tunes the YOLOv5 [1] model, pre-trained on the COCO dataset [2].

To fine-tune the model, we trained the YOLOv5x6 checkpoint, available in the Yolo-v5 repository, for 200 epochs with a batch size of 60 images, using Stochastic Gradient Descent optimization. We use a learning rate of 0.05 with a weight decay of 0.00036 and momentum of 0.843. During training, all images were resized to 640×640 pixels. Additionally, data augmentation techniques such as translation, flipping, and scaling were applied. We used a Quadro RTX 8000 GPU with 48GB of VRAM to

fine-tune the model. All training parameters and training logs are available on the repository of this work.

During inference, images retained their original sizes. Unlike standard object detection, our panel extraction method does not output overlapping bounding boxes, as scientific image panels do not overlap. Consequently, predicted panels do not share areas with other panels.

To achieve non-overlapping results, we employed two post-processing techniques. First, non-max suppression (NMS) was applied with an intersection-over-union threshold of 0.4 to identify overlapping boxes, retaining only the box with the highest confidence score from each set of overlaps. Second, we merged any remaining overlapping predicted boxes of the same panel type (e.g., microscopy panels) to ensure non-overlapping panels in the output.

Image Provenance Parameter Analysis

To investigate the image provenance parameters, we opted to use the Extended SPP (v1) dataset during the experiments. This choice was based on the dataset's balance between having an adequate number of distractors while maintaining a manageable size. We avoided using the Extended SPP (v2) dataset as it would have significantly increased the time required to complete the experiments.

In the following, we explain each parameter and also present a set of experiments that motivated our parameter choice. Throughout these experiments, a specific set of parameters remained constant, and we solely varied those parameters under analysis. When examining a particular parameter, the remaining parameters were fixed at predetermined values (all determined after an exploratory investigation):

- Top-K = 400;
- Minimum Shared Area = 0.01;
- Minimum Interest Point Matching = 20;
- Maximum Processing Queue Size = 300.

Top-K is employed in the content retrieval phase to determine the number of the most similar images to a probe to be included in the processing queue. A small value of K can decrease the likelihood of detecting reused panels, while a high K may introduce significant overhead to the pipeline, as the probe and lower-ranked items might have substantially different content. Fig. S1 expresses our experiment, in which we varied K with values 40, 200, and 400. The results indicate that a small value of K lowers the performance of our method, while a higher value of K increases its performance. When analyzing $K = 200$ and $K = 400$, we noticed a slight increase in the *Content Pairing* metric of *Document Evaluation*. All the other metrics were very similar using both parameters. We preferred to choose $K = 400$ due to this slight increase in performance, with a small impact on the method runtime.

Top-L is employed to determine the number of elements included in the processing queue based on the ranked similarity of a matching panel. In our case, L is set to 40, which corresponds to 10% of the top-K retrieved items. The purpose of a second-tier retrieval is to introduce more diversity into the processing queue. Due to the vast editing possibilities, two panels with shared content could appear visually different and not be included in the top-k similarity rank of one another. By adding this second-tier retrieval rank, we increase the chances of comparing a not-so-similar panel from the

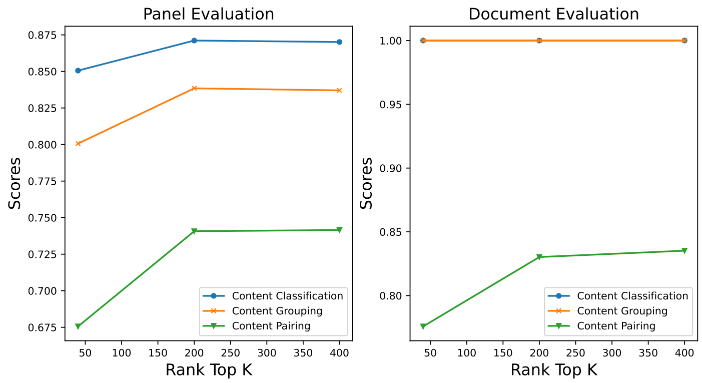

Fig S1. Top-K parameter analysis. The performance of the method improves with a higher number of K.

probe, thereby raising the probability of finding two matching images. While the processing queue is set to 300 elements, it is important to note that this queue only includes pairs of images that have not been processed previously, keeping in mind that each probe is processed in parallel (i.e., simultaneously). Thus, even if the number of retrieved items K is higher than 300, there may be a higher number of redundant pairs that have already been or are being analyzed in parallel, which will be ignored. For example, if Px is the image probe and Py is an image within the top-K ranking of Px, when analyzing Py, there is a high probability that Px will also be within the top-K ranking of Py. Because of that, Px will be discarded from the top-k rank of Py since the pair (Px, Py) has already been processed.

The presence of overlapping processing pairs across various rankings motivated an adjustment in the parameter K, allowing it to surpass the processing queue's maximum size. Additionally, we introduced a second-tier retrieval rank to the queue, intending to enhance diversity within the queue and increase the chances of identifying new matches.

Minimum Shared Area defines the smallest shared area that two panels must have to be considered a reuse. This parameter varies from 0.0 to 1.0, qualifying the percentage of the shared area between an image pair. Setting it to 0.0 implies that any retrieved item from the top-K will be considered as a possible reuse, while setting it to 1.0 implies that only exact matches will be considered. It is important to note that some panels may contain annotations like arrows and letters, and without a minimum shared area, there might be many false positive matches due to these annotations. During our analysis, we varied the minimum shared area between a pair of images to 1%, 5%, and 10%, as shown in Fig. S2. Our experiment reveals that an increment in the minimum required area reduces the pairing scores' performance, which measures a method's efficacy in accurately linking reused elements. By analyzing the precision and recall of the method (Fig. S3 and Fig. S4), a high value of the "minimum shared area" parameter renders the method excessively conservative in confirming a match, resulting in the exclusion of valid positive matches found on a relatively small shared area. In other words, an elevated "minimum shared area" provides a high precision score at the expense of a decreased recall.

Minimum Interest Point Matching defines the minimum number of interest points located by SIFT that must undergo geometric matching. This parameter is crucial because too few interest points can lead to an increased number of false positives. We conducted experiments using three values for this parameter: 10, 20, and 50. This

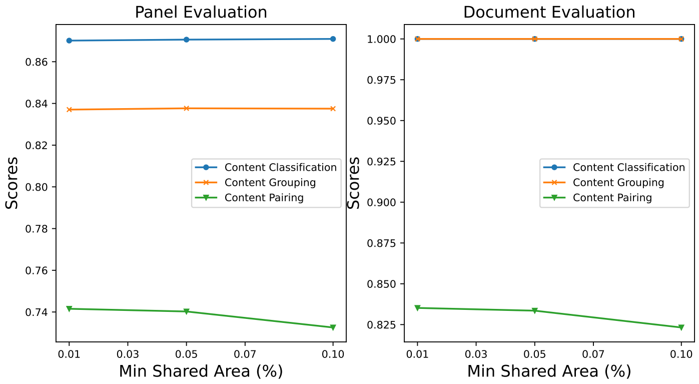

Fig S2. Analysis of the percentage of Minimum Shared Area between two images for identifying problematic reuse.

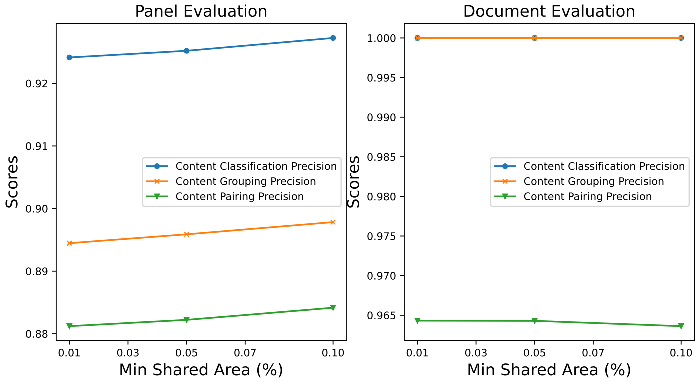

Fig S3. Precision analysis of the percentage of Minimum Shared Area between two images for identifying problematic reuse.

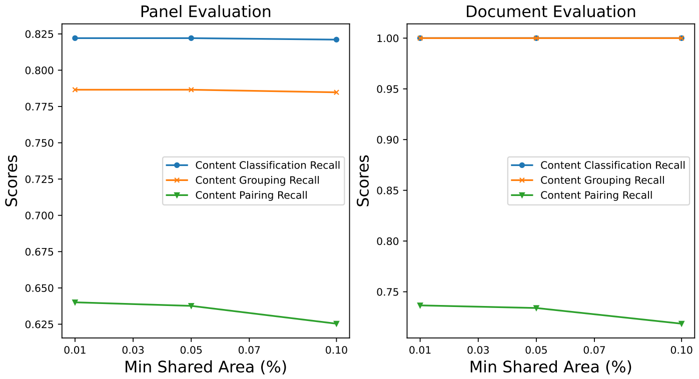

Fig S4. Recall analysis of the percentage of Minimum Shared Area between two images for identifying problematic reuse.

evaluation enabled us to gauge the impact of the number of interest points on method performance and find an equilibrium between accuracy and false positives.

In a similar manner to the minimum area experiment, an increase in the minimum number of interest points leads to a decrease in method performance. As observed in Fig. S6 and Fig. S7, when setting the parameter to a high number of interest points, the solution prioritizes precision over recall. Through our experimentation, we have

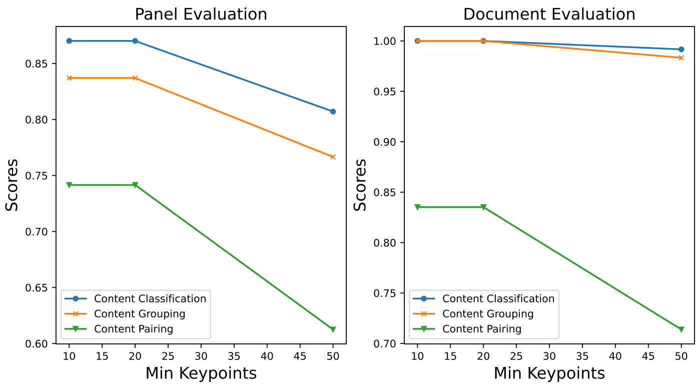

Fig S5. Analysis of Minimum Interest Point Matching between two images to assess problematic reuse.

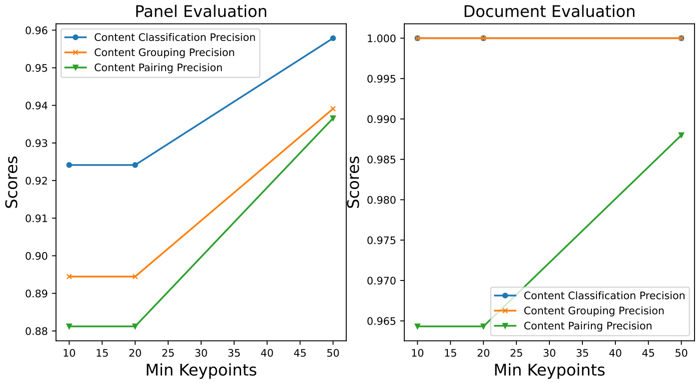

Fig S6. Precision Analysis of Minimum Interest Point Matching between two images to assess problematic reuse.

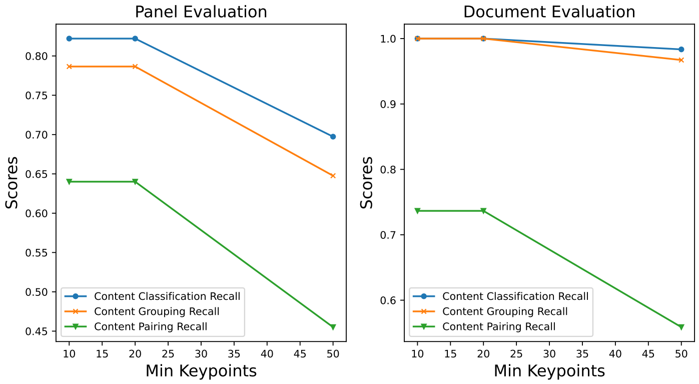

Fig S7. Recall Analysis of Minimum Interest Point Matching between two images to assess problematic reuse.

determined that setting this value to 20 interest points strikes a balance between precision and recall while apparently considering enough points to prevent undesired matches on other images not included in the experiment.

Maximum processing queue size plays a crucial role in the scalability of our method, as it significantly reduces the number of elements that need to be compared,

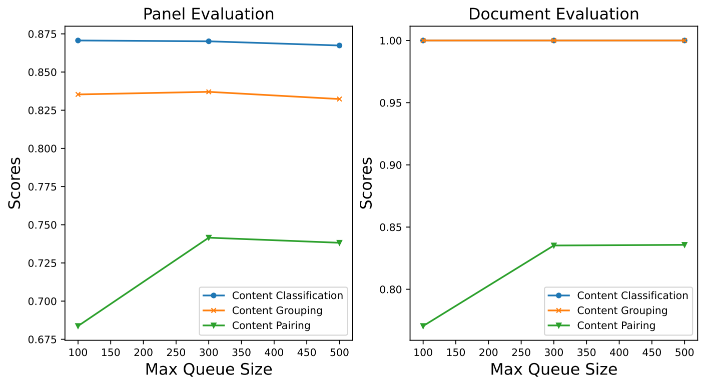

Fig S8. Evaluation of the maximum number of analyzed candidates per probe, denoted by the processing queue size.

from all-vs-all to a limited but well-selected number of comparisons. Fig. S8 shows our experiments when considering a processing queue with a maximum size of 100, 300, and 500. As depicted in that figure, the optimal results were obtained with a queue size of 300. Our analysis of these findings suggests that when an excessively large number of distractors is introduced into the processing queue, the likelihood of matching annotation distractor elements also increases, despite our efforts to mitigate this by setting a *minimum number of interest points* and *minimum shared area* when analyzing a match. Conversely, a small processing queue may not provide the method with the capacity to identify reused pairs effectively, as expressed by the lower performance of a queue with size 100. Therefore, a queue size of 300 strikes the right balance in this regard.

References

1. Jocher G, Stoken A, Chaurasia A, Borovec J, NanoCode012, TaoXie, et al.. ultralytics/yolov5: v6.0 - YOLOv5n 'Nano' models, Roboflow integration, TensorFlow export, OpenCV DNN support; 2021. Zenodo.

2. Lin TY, Maire M, Belongie S, Hays J, Perona P, Ramanan D, et al. Microsoft COCO: Common Objects in Context. In: Computer Vision - ECCV 2014. Springer International Publishing; 2014. p. 740–755.
